# LacSubPred: predicting subtypes of Laccases, an important lignin metabolism-related enzyme class, using *in silico *approaches

**DOI:** 10.1186/1471-2105-15-S11-S15

**Published:** 2014-10-21

**Authors:** Tyler Weirick, Sitanshu S Sahu, Ramamurthy Mahalingam, Rakesh Kaundal

**Affiliations:** 1 National Institute for Microbial Forensics & Food and Agricultural Biosecurity (NIMFFAB), Oklahoma State University, Stillwater, Oklahoma, 74074, USA; 2 Department of Biochemistry & Molecular Biology, Oklahoma State University, Stillwater, Oklahoma, 74074, USA; 3 Bioinformatics Facility, Department of Botany & Plant Sciences, Institute for Integrative Genome Biology (IIGB), University of California, Riverside, California, 92521, USA

**Keywords:** Bioenergy, Lignin degradation / synthesis, Laccases, Machine Learning, Classification, Bioinformatics, Unsupervised learning

## Abstract

**Background:**

Laccases (E.C. 1.10.3.2) are multi-copper oxidases that have gained importance in many industries such as biofuels, pulp production, textile dye bleaching, bioremediation, and food production. Their usefulness stems from the ability to act on a diverse range of phenolic compounds such as o-/p-quinols, aminophenols, polyphenols, polyamines, aryl diamines, and aromatic thiols. Despite acting on a wide range of compounds as a family, individual Laccases often exhibit distinctive and varied substrate ranges. This is likely due to Laccases involvement in many metabolic roles across diverse taxa. Classification systems for multi-copper oxidases have been developed using multiple sequence alignments, however, these systems seem to largely follow species taxonomy rather than substrate ranges, enzyme properties, or specific function. It has been suggested that the roles and substrates of various Laccases are related to their optimal pH. This is consistent with the observation that fungal Laccases usually prefer acidic conditions, whereas plant and bacterial Laccases prefer basic conditions. Based on these observations, we hypothesize that a descriptor-based unsupervised learning system could generate homology independent classification system for better describing the functional properties of Laccases.

**Results:**

In this study, we first utilized unsupervised learning approach to develop a novel homology independent Laccase classification system. From the descriptors considered, physicochemical properties showed the best performance. Physicochemical properties divided the Laccases into twelve subtypes. Analysis of the clusters using a *t*-test revealed that the majority of the physicochemical descriptors had statistically significant differences between the classes. Feature selection identified the most important features as negatively charges residues, the peptide isoelectric point, and acidic or amidic residues. Secondly, to allow for classification of new Laccases, a supervised learning system was developed from the clusters. The models showed high performance with an overall accuracy of 99.03%, error of 0.49%, MCC of 0.9367, precision of 94.20%, sensitivity of 94.20%, and specificity of 99.47% in a 5-fold cross-validation test. In an independent test, our models still provide a high accuracy of 97.98%, error rate of 1.02%, MCC of 0.8678, precision of 87.88%, sensitivity of 87.88% and specificity of 98.90%.

**Conclusion:**

This study provides a useful classification system for better understanding of Laccases from their physicochemical properties perspective. We also developed a publically available web tool for the characterization of Laccase protein sequences (http://lacsubpred.bioinfo.ucr.edu/). Finally, the programs used in the study are made available for researchers interested in applying the system to other enzyme classes (https://github.com/tweirick/SubClPred).

## Background

Laccases (EC 1.10.3.2) are the largest sub-group of multi-copper oxidases which includes ascorbate oxidases (EC 1.10.3.3), ferroxidases or ceruloplasmins (EC 1.16.3.1) and nitrate reductases (EC 1.7.2.1). Laccases were first discovered in the sap of the Japanese lacquer tree *Rhus vernicifera*. Since then they have been found in many taxa including plants, fungi, bacteria, and metazoa. Laccases are involved in a diverse range of cellular activities such as lignin degradation, lignin biosynthesis, pigment production, plant pathogenesis, melatonin production, spore coat resistance, morphogenesis and detoxification of copper [1-5]. Laccases are also widely used for industrial purposes. For example, Laccases are in paper and pulp, textile, and petrochemical industries for detoxification of industrial effluents [6]. In medicine, Laccases are used for certain medical diagnostics and as catalysts for the manufacture of anti-cancer drugs [6]. They are also used for environmental remediation of herbicides, pesticides and as explosives in soil and cleaning agents for certain water purification systems. In commercial products, they are found in cosmetics, denim bleaching, wine and beer stabilization, fruit juice processing, color enhancement of tea and even baking [6,7]. Laccases are popular in industry for a number of reasons. They are better for the environment, and have fewer non-specific reactions than conventional oxidation technologies. Many Laccases are extracellular enzymes which makes their purification simple. Compared with other oxidative enzymes, these are easier to use as they catalyze reactions with molecular oxygen and do not need reactive oxygen species catalysis [6,8]. Currently, fungal Laccases comprise most widely studied and commercially used Laccases. However, there is much interest in bacterial Laccases also due to their higher temperature stability and ability to operate at different pHs than fungal Laccases. Generally, Laccases are composed of dimeric or tetrameric glycoproteins with each monomer containing a copper containing site. These copper sites may be one of three types: Type-1 or blue copper, Type-2 or normal copper, and Type-3 or coupled-dinuclear centers. These copper binding motifs have been shown to be highly conserved across all Laccases, with a trend towards greater similarity in the N and C terminal domains as these are the copper containing domains. It has been noted that the size of the central binding pockets are larger in bacterial Laccases than in fungal or plant Laccases. These copper binding sites yield significant differences in conserved residues for Laccases of bacteria, fungi, and plants [9].

### Fungal Laccases

Fungal Laccases comprise the bulk of experimentally studied Laccases. They occur in many fungal species and are thought to play important roles in morphogenesis, fungal-plant interactions, stress defense, pigment production, and lignin degradation. While typically studied with respect to biomass degradation, most fungi found producing several isoenzymes of different types, enzymatic or physical properties, and expression levels. These can vary even more between species [8]. For example, it has been reported that one of the most efficient lignin degraders, *Phanerochaete chrysosporium *produces a Laccase different than other efficient lignin degrading fungi [10]. While most Laccases are extracellular enzymes, many fungal taxa produce intracellular Laccases [8] also. This is especially interesting when compared with enzymes of similar function such as lignin peroxidases which are strictly extracellular. It is speculated that the cellular localization of Laccases may be connected their function and substrate ranges. This hypothesis still remains elusive due to the majority of studied fungal Laccases coming from wood-rotting basidiomycetes. The enzymatic properties of fungal Laccases vary greatly such as temperatures vary from 25-80° C, pH optimums: 2,2'-azino-bis(3-ethylbenzothiazoline-6-sulphonic acid) (ABTS) from 2.0-5.0, 2,6-dimethoxyphenol (DMP) from 3.0-8.0, guaiacol from 3.0-7.0, and syringaldazine from 3.5-7.0. Similarly, K_m _(µM) ranges vary a lot such as: ABTS from 4-770, DMP from 26-14720, Guaiacol from 4-30000, syringaldazine from 3-4307. Also K_cat _(S^-1^) vary in a broad range as: ABTS from 198-350000, DMP from 100-360000, Guaiacol from 90-10800 and syringaldazine from 16800-28000. These properties can further be altered by glycosylation.

### Plants Laccases

Traditionally plant Laccases were considered to be only extracellular enzymes involved in the radical-based lignin polymerization. However, a high degree of divergence among Laccases within a single plant species has been observed, such as ryegrass which contains 25 different Laccase genes. Also, it is reported that Laccases lack N-terminal signal peptides for secretion but have signals targeting to other cellular components such as the endoplasmic reticulum or peroxisomes. Another study on poplars showed that Laccase repression had no effect on lignin production. Despite the evidence for novel functions and many known functions in other taxa, the grouping of plant Laccases still remain elusive [11].

### Bacterial Laccases

Bacterial Laccases are known to be widespread in prokaryotes; however, only few have been experimentally characterized. To date, bacterial Laccases have been found mostly to be involved in lignin degradation, catabolism of phenolic compounds, cell pigmentation, morphogenesis, and copper defense [12-14]. The best studied bacterial Laccase is CotA and endospore coat protein from *Bacillus subtilis *which produces a melanin like pigment. This enzyme has generated high amounts of interest due to its extremely high temperature stability. Bacterial Laccases are also unique due to the lack of cellular partitions in prokaryotes. The reactions catalyzed by Laccases can produce quinones and semiquinones as by-products, which are powerful inhibitors of the electron transport change [5].

### Other Laccases

In metazoan, Laccases exist in mammals as well as invertebrates. The roles of Laccases in mammals do not appear to be well understood, however, insect Laccases are known to be involved in cuticle formation [12]. Cuticle tanning also known as sclerotiziation and pigmentation is the process through which proteins in the exoskeleton are conjugated. This causes the exoskeleton to become insoluble, harder, and darker.

### Classification of Laccases: current view

Laccases are currently classified as part of a larger classification scheme for multi copper oxidases [15,16]. This is based on multiple sequence alignments and seems to classify by taxonomical association. The current classification system i.e. "The Laccase Engineering Database" (LccED), classifies multi copper oxidases into eleven classes: basidiomycetes Laccases, ascomycete Laccases, insect Laccases, fungal pigment MCOs, Fungal ferroxidases, fungal and plant ascorbate oxidases, plant Laccases, bacterial CopA proteins, bacterial bilirubin oxidases, bacterial CueO proteins, and SLAC homologs.

### Machine learning-based classification systems

As discussed above, the current classification system for Laccases largely follow species taxonomy rather than substrate ranges, enzyme properties, or specific function. Although it has been observed that individual Laccases often exhibit distinctive and varied substrate ranges, and have different functions based on distinguishing pH values among different taxa. We hypothesize that a descriptor-based computational prediction system could be developed to generate a homology-independent classification system for better describing the functional properties of Laccases. In a previous study on feruloyl esterases (EC 3.1.1.73), an unsupervised learning approach was used to create a novel homology independent classification system for this enzyme class. Various bioinformatics tools were used to validate the identified classes [17]. In the present study, we followed a two-way computational strategy to identify and classify various Laccase subtypes by developing a python command line-based implementation of the unsupervised and supervised learning approaches, respectively. Further, we implemented our prediction models as a web-based prediction server to classify novel Laccase subtypes. The tool could be useful to the biofuel researchers and industry as well.

## Methods

### Dataset generation

**Figure 1:**
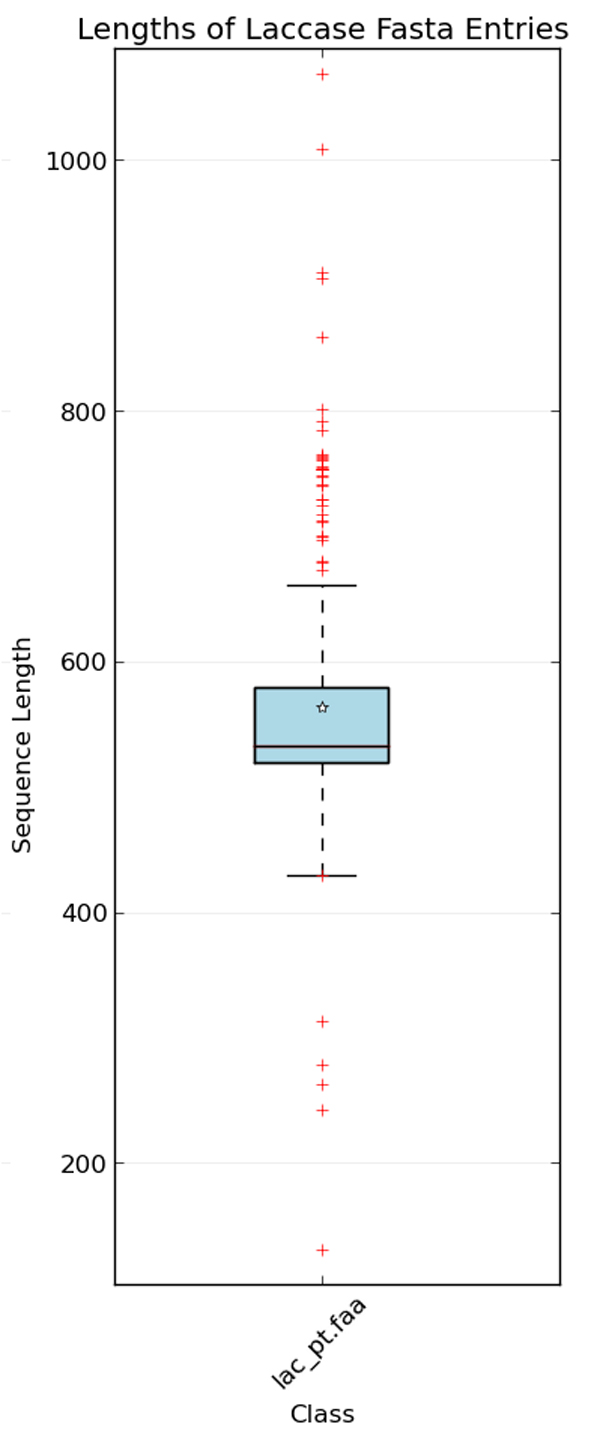
**Distribution of protein sequence lengths contained within the initial dataset used in the study**. The box represents the first and third quartiles, the band the second quartile, the star is the median, and the whiskers are 1.5 the interquartile range. This was done to identify sequences with significant differences in length. The outliers on the top and bottom were manually reviewed.

Alternate names for Laccases were found via cross referencing with the KEGG database (http://www.kegg.jp/dbget-bin/www_bget?ec:1.10.3.2). To search for Laccase sequences, we combine these names to start as a basic query. Sequences with protein or transcript level evidence were selected to ensure high quality data as well as avoid potentially mislabeled multi-copper oxidases. Then we search UniprotKB for Laccase sequences using some search terms as listed in Table 1. Using the "browse by" option on Uniprot's GUI the query was checked for possible contaminating sequences. The contaminant sequences were filtered out using NOT conditions (see Table 1). Finally, 329 protein sequences are collected with average sequence length above 200 residues. To further validate the quality of the datasets the protein descriptions of the data were analyzed with the text clustering functionality in Google-Refine version 2.5. A significant variation was found in the protein descriptions but no cases of contamination were found. As a final check of data quality, the lengths of the sequences were calculated and plotted on a bar graph shown in Figure 1. Sequences containing non-standards/ambiguous characters were removed from the data set.

**Table 1:** Search terms used for collecting Laccases-related enzymes from UniProtKB database.

Basic Query	Final query
(name:Laccase OR name:"urishiol oxidase" OR name:"urushiol oxidase" OR name:"p-diphenol oxidase" OR ec:1.10.3.2) AND (existence:"evidence at protein level" OR existence:"evidence at transcript level") AND fragment:no	(name:Laccase OR name:"urishiol oxidase" OR name:"urushiol oxidase" OR name:"p-diphenol oxidase" OR ec:1.10.3.2) AND (existence:"evidence at protein level" OR existence:"evidence at transcript level") AND fragment:no AND NOT name:"Catechol oxidase" AND NOT ec:1.10.3.1 AND NOT ec:1.10.3.3

### Feature representation of Laccase proteins

It is important to extract better features of protein sequences to improve the performance of the machine learning method. We used several features such as amino acid composition (AAC), Conjoint Triad (CT), Composition-Transition-Distribution (CTD), Dipeptide composition (DIPEP), Geary autocorrelation descriptors, Moran autocorrelation, Moreau-Broto autocorrelation, physicochemical properties and a composite vector of amino acid composition and physicochemical properties.

### Amino acid composition (AAC)

Each protein sequence is represented as a 20-dimensional feature vector with each element corresponding to the percentage of one of the twenty amino acids [18]. For a given protein sequence *x*, let the function f(*x_i_*) represent the occurrence of the 20 standard amino acids. Thus, the composition of the amino acids Px in the given sequence can be represented as,

(1) $$P\left(x\right)=\left[P_{1}\left(x\right),P_{2}\left(x\right),...,P_{20}\left(x\right)\right]$$

where P(*x_i_*) is given as,

(2) $$P\left(x_{i}\right)=\frac{f\left(x_{i}\right)}{\sum_{i=1}^{20}f\left(x_{i}\right)}i=1,2,3,\ldots 20$$

### Dipeptide composition (DIPEP)

Dipeptide sequence composition is similar to amino acid composition. However, it considers the percentages of dipeptides occurring in a given protein sequence [18]. Thus, the composition of each dipeptide is given as,

(3) $$P\left(x_{i},x_{j}\right)=\frac{f\left(x_{i},x_{j}\right)}{\sum_{i=1}^{20}\sum_{j=1}^{20}f\left(x_{i},x_{j}\right)}i,j=1,2,3\ldots ...20$$

where $P\left(x_{i},x_{j}\right)$ is the fraction of number of instances of a specific dipeptide $f\left(x_{i},x_{j}\right)$ and the total number of all dipeptides.

### Conjoint triad (CT)

In conjoint triad, in addition to amino acid composition it considers the sequence order effect [19]. It is calculated by grouping the 20 standard amino acids into 7 groups based on physical and chemical similarity [(A,G,V), (I,L,F,P),(Y,M,T,S), (H,N,Q,W), (R,K), (D,E), (C)]. Triads are made from all combinations of three amino acids of these groups, resulting in a vector length of 343 (7 × 7 × 7). Thus, a protein sequence is represented as,

(4) $$P\left(x_{i},x_{j},x_{k}\right)=\frac{f\left(x_{i},x_{j},x_{k}\right)}{\sum_{i=1}^{7}\sum_{j=1}^{7}\sum_{j=1}^{7}f\left(x_{i},x_{j},x_{k}\right)}i,j=1,2,3\ldots ...20$$

where $f\left(x_{i},x_{j},x_{k}\right)$ is the number of occurrences of a specific triad and $\overset{7}{\underset{i=1}{\sum }}\overset{7}{\underset{j=1}{\sum }}\overset{7}{\underset{j=1}{\sum }}f\left(x_{i},x_{j},x_{k}\right)$ is the number of all triads [19].

### Composition-transition-distribution (CTD)

In this representation three local descriptors, Composition (C), Transition (T) and Distribution (D) are used in combination to construct the feature vector. These descriptors are based on the variation of occurrence of functional groups of amino acids within the primary sequence of protein [20]. Thus, before computing this feature the twenty amino acids are clustered into seven functional groups based on the dipoles and volumes of the side chains [19]. The composition descriptor computes the occurrence of each amino acid group along the sequence. Transition represents the percentage frequency with which amino acid in one group is followed by amino acid in another group. The distribution feature reflects the dispersion pattern along the entire sequence by measuring the location of the first, 25, 50, 75 and 100% of residues of a given group. Hence, total 63 features (7 composition, 21 transition and 35 distribution) are constructed to represent a protein.

### Autocorrelation feature vectors

Autocorrelation features describe the level of correlation between two protein sequences in terms of their specific physicochemical property, which are defined based on the distribution of amino acid properties along the sequence. There are 8 amino acid properties used for deriving autocorrelation descriptors.

### Moran autocorrelation

The Moran autocorrelation (MAC) descriptor of a protein is defined as:

(5) $$D_{MAC}\left(d\right)=\frac{\frac{1}{N-d}\sum_{j=1}^{N-d}\left(P_{j}-\bar{P}\right)\times \left(P_{j+d}-\bar{P}\right)}{\frac{1}{N}\sum_{j=1}^{N}{\left(P_{j}-\bar{P}\right)}^{2}}$$

where *N *is the length of the protein sequence, d = 1,2,......30 is the distance between one residue and its neighbors, *P_j _*and *P_j+d _*are the properties of the amino acid at positions *j *and *j+d *respectively. $\bar{P}=\overset{N}{\underset{j=1}{\sum }}\frac{P_{j}}{N}$ is the average of the considered property P along the sequence.

### Geary autocorrelation

Geary autocorrelation (GA) descriptor of a protein is defined as:

(6) $$D_{GA}\left(d\right)=\frac{\frac{1}{2\left(N-d\right)}\sum_{j=1}^{N-d}{\left(P_{j}-P_{j+d}\right)}^{2}}{\frac{1}{N-1}\sum_{j=1}^{N}{\left(P_{j}-\bar{P}\right)}^{2}}$$

$\bar{P}$, N, P_j _and P_j+d _are defined in the same way as above.

### Moreau-Broto autocorrelation

Moreau-Broto autocorrelation (MBA) descriptor of a protein is defined as:

(7) $$D_{MBA}\left(d\right)=\overset{N-d}{\underset{j=1}{\sum }}P_{j}\times P_{j+d}$$

$\bar{P}$, N, P_j _and P_j+d _are defined in the same way as above.

### Physicochemical properties

Physicochemical properties of amino acids have been used successfully in numerous prediction tools [18]. In this study, we grouped the amino acids of a protein into classes based on some physicochemical properties. Also the theoretical pI, molecular weight, and length of the protein are used in the feature vector. The non-composition based values are divided by the length or mass on the protein titan in order to provide values between one and zero. Molecular weights were calculated by adding the weights of the each amino acid in the sequence in a suitable way related to their chemical activity. A detailed description of these properties is provided in Table 2.

**Table 2:** Physicochemical properties used to represent a protein for Laccase subclass prediction.

**Sr. No**.	Physicochemical property	Amino Acids	# features
1	log10(molecular weight)/7.0	-	1
2	log10(Sequence Length)/5.0	-	2
3	% Charged Residues	DREKH	3
4	% Hydrophilic and neutral	NQSTY	4
5	% basic polar/positively charged	HKR	5
6	% acidic or negatively charged	DE	6
7	% aliphatic	AGILV	7
8	% aromatic	FWY	8
9	% small	DNT	9
10	% tiny	AGPS	10
11	% large	FRWY	11
12	% hydrophobic and aromatic	WF	12
13	% hydrophobic and neutral	ACGILMFPWV	13
14	% amidic	NQ	14
15	% cyclic	P	15
16	% hydroxylic	ST	16
17	% contains sulfur	CM	17
18	% H-bonding	CWNQSTYKRHDE	18
19	% acidic and amide	DENQ	19
20	% ionizable	DEHCYKR	20
21	% sulfur bonding	C	21
22	% pI	-	22
23	Molecular weight/ 4000000.0	-	23
24	Sequence length / 38000.0	-	24

### Split amino acid composition

Split amino acid composition aims to capture information about signal peptides at their N- or C-terminal region. The amino acid composition of the N-terminal region, Center, and C-terminal region are computed and then concatenated together. The N- and C- terminal regions are the first and last 25 amino acids in the sequence. Thus a protein sample is represented as a 60 element vector as,

(8) $$P\left(x\right)=\left[AAC_{N-terminal}AAC_{Center\;region}AAC_{C-erminal}\right]$$

### Unsupervised classification

Unsupervised learning organizes the data based on the similarity patterns between them. In this study, clustering was used to group the data into classes sharing same type of similarity not found in other classes. We followed the similar methodology as outlined in the paper [17]. We first used self- organizing map (SOM) to identify the possible number of groups in the dataset and used that information in *k*-means clustering to divide them in different clusters.

### Self-organizing maps (SOM)

SOMs are a type of artificial neural networks used in unsupervised learning to produce low dimensional discrete representations of the vector space represented by some training data [21]. The discrete elements in SOMs are called nodes or neurons. It has been used widely in bioinformatics and computational biology mostly for tasks such as finding gene expression patterns and protein classification [22,23]. The SOM map contains *m *neurons, where each contains a d-dimensional prototype vector with d as the dimensions of the input vectors. First, initial values were given to each prototype vector. When training begins a vector 'x' from the input data is randomly chosen. The distances from 'x' to the prototype vectors are computed and the neuron closest to 'x' or best matching unit (BMU) is selected. The radius of the neighborhood of the DMU is calculated, any neurons found within the radius are deemed neighbors. The neighbor's prototype vector is adjusted to be more similar to the input vector. This procedure was then repeated for certain iterations (N) [21]. In this study, SOM of multiple dimensions were studied and N was 10,000 for all dimensions. For the SOM implementation, we used an open source machine learning package 'Orange.py' which is freely available at http://orange.biolab.si[24].

### K-means clustering

K-means clustering is a class of unsupervised learning algorithms which group input data set into '*k*' parts or clusters [25] based on similarity measure. K-means is one of the oldest and simplest clustering methods, however still remains a useful tool for cluster analysis. It scales well to large data sets and medium numbers of clusters, however, has the drawback of needing to specify the number of clusters expected. The basic *k*-mean algorithm begins by initializing *k *cluster centers (centroids) and iterating to minimize the average distances between centroids and their cluster members. The data which are close to any cluster centroid belong to that cluster. The centroids were pre-computed using the neurons from the SOM. In this study, an open source machine learning library 'Sci-Kit Learn' was used to implement the *k*-means clustering method [26].

### SOM for finding *K *number and centroid locations for *K*-means clustering

In this study, first an SOM network computed containing N neurons and calculates the Davies-Bouldin index (DBI) of the map treating the neurons as clusters. Then, (N) × (N-1) prototype maps were created by making all combinations of each neuron with the other neurons. The DBI is computed for all prototype maps, and the prototype map with the lowest DBI is selected. If the DBI of this map is lower than the current map the map is changed to other prototype map and the previous steps are repeated until no prototype map with a lower DBI can be found. This reduces the size of the map by one each iterations with the final number of neurons being used as the k value for k-means clustering and the cluster centroids are computed from the vectors belonging to each neuron. The efficiency of k-means clustering is measured using the difference between the inter-cluster and intra-cluster variance and the Davies-Bouldin index. As SOM find the clusters in random fashion, to get the optimum number of clusters, the clustering procedure was run 500 times for each vector type. The optimum number of clusters was chosen by selecting the cluster from the most often occurring cluster number with the largest intercluster and intracluster difference and smallest DBI.

### Davies-Bouldin index (DBI)

The DBI is a metric for evaluating overall quality of a given set of clusters originally developed to aid in determining the optimum number of clusters within a dataset [27]. Minimization of the DBI of the clusters within a dataset seems to generally indicate natural partitions of data sets. However, it should be noted that this is a heuristic approach and good values do not always indicate the best clustering arrangement. DBI of a clustering approach is defined as,

(9) $$DB\equiv \frac{1}{N}\overset{N}{\underset{i=1}{\sum }}D_{i}$$

where D_i _is the worst case scenario of all values of R_i,j_,

(10) $$D_{i}\equiv \underset{j:i\neq j}{\text{max}}R_{i,j}$$

R_i,j _is a measure of the clustering quality, defined as

(11) $$R_{i,j}\equiv \frac{S_{i}+S_{j}}{M_{i,j}}$$

The measure of scatter (S) within a given cluster i, is defined as

(12) $$S_{i}=\sqrt[{q}]{\frac{1}{T_{i}}\overset{T_{i}}{\underset{j=1}{\sum }}{\left|X_{j}-A_{i}\right|}^{q}}$$

where X_j _is a n-dimensional feature vector assigned to the cluster C_i _and q was kept as two and M_i,j _is a measure of separation between two clusters defined as

(13) $$M_{i,j}=A_{i}-{A_{j}}_{p}$$

where A_i _is the centroid of cluster C_i _containing samples X_1_,X_2_......X_k _and computed as,

$$A_{i}=\frac{X_{1}+X_{2}+X_{3}+\ldots +X_{k}}{k}$$

### Intra-cluster variance

Intra-cluster variance was calculated using the Euclidean distances between the points in the cluster and the centroid of the cluster.

### Inter-cluster variance

Inter-cluster variance was calculated using the Euclidean distance between the centroids of the clusters.

### Co-occurrence matrix analysis

The cluster numbers returned from the clustering approach is arbitrary which presents a unique problem when trying to access the similarity between runs. Thus, to assess the consistency of belonging of samples in a particular group, a co-occurrence matrix was generated to show the number of times a given data sample in one group occurred with other groups. The higher the numbers of data samples occurring together, the more consistency the clusters in various runs.

### Support vector machine (SVM)

SVMs are a class of supervised learning algorithms based on the optimization principle from statistical learning theory [28,29]. Support vector machines have been used widely in computational biology in diverse topics such as subcellular localization [18,30-32], protein function prediction [33], secondary structure prediction [34], disease forecasting [35]. SVMs solve classification problems by calculating a hyperplane that separates the training data with a maximum margin. For multi-class classification the classification is transformed into a series of binary classifications. There are numerous strategies for handling a multi-class problem separated into binary classifications and in this study the one-versus-rest approach was used. The SVM Classifiers were developed using the SVM_Light package (https://github.com/daoudclarke/pysvmlight), which is an open source package for SVM implementation [36]. In a preliminary study, the RBF kernel was found to perform best. Therefore, we used RBF kernel in all our SVM classifiers.

### Performance evaluation parameters

To assess the performance of the developed models, we used a five-fold cross validation test on the training dataset and then tested the models in an independent test. In a five-fold cross-validation procedure, the original sample is randomly partitioned into five equal size subsamples. Of the five subsamples, a single subsample is retained as the validation data for testing the model, and the remaining four subsamples are used as training data. The cross-validation process is then repeated 5 times (the *folds*), with each of the 5 subsamples used exactly once as the validation data. The results from the five-folds are then averaged to produce a single estimation. The performance is measured by the parameters such as overall sensitivity, specificity, precision, Matthews Correlation Coefficient (MCC) and average accuracy. These parameters are defined as follows:

(i) Sensitivity or coverage of positive examples: It is the percent of positive samples correctly predicted,

(14) $$\text{Sensitivity}\left(S_{n}\right)=\frac{TP}{TP+FN}$$

(ii) Specificity or coverage of negative examples: It is percent of negative samples correctly predicted as positive,

(15) $$\text{Specificity}\left(\text{Sp}\right)=\frac{\text{TN}}{\text{TN}+\text{FP}}\times 100$$

(iii) Accuracy: It is the percentage of correctly predicted samples,

(16) $$\text{Accuracy}\left(\text{Acc}\right)=\frac{\text{TP}+\text{TN}}{\text{TP}+\text{FN}+\text{FP}+\text{FN}}\times 100$$

(iv) Error rate: It is the total percentage of incorrect predictions is calculated as

Error rate (ER) = $\text{Error}\;\text{rate}\left(\text{ER}\right)=\frac{\text{FP}+\text{FN}}{\text{TP}+\text{FN}+\text{FP}+\text{FN}}\times 100$ (17)

(v) Precision: It is the percentage of positive PPIs those are correct identified true prediction,

(18) $$\text{Precision}=\frac{TP}{TP+FP}\times 100$$

(vi) Matthew's correlation coefficient (MCC): it is considered to be the most robust parameter of any class prediction method. MCC equal to 1 is regarded as perfect prediction while 0 for completely random prediction.

(19) $$\text{MCC}=\frac{\left(\text{TP}\times \text{TN}\right)-\left(\text{FP}\times \text{FN}\right)}{\sqrt{\left(\text{TP}+\text{FP}\right)\left(\text{TP}+\text{FN}\right)\left(\text{TN}+\text{FP}\right)\left(\text{TN}+\text{FN}\right)}}$$

where true positive TP) is the numbers of positive samples that are predicted correctly; false negative (FN) is the number of positive samples that are predicted to be negative; false positive (FP) is the number of negative samples that are predicted positive and true negative (TN) is the number of negative samples that are predicted correctly as negative.

### Feature scaling

To have knowledge of most relevant features for classification of Laccase types, a feature scaling approac is conducted. Feature scaling was performed using univariate feature selection using the functions provided by Sci-Kit Learn using the program scale_features.py[26]. Univariate feature selection implemented by considering each element of the descriptor vectors independent from one another and ranking them based on their occurrence between classes.

### Domain map and phylogenetic trees construction

The program doMosaic was used to create domain maps for visualization of the domains in the initial data and newly generated classes [37]. Interproscan was used to get the information about the domains in the Laccases [38]. To show the relationship between Laccase samples, a phylogenetic tree was generated with the cleaned dataset using Clustal Omega version 1.0.3 [39]. Dendroscope version 3.2.10 was used for the visualization of the tree.

## Results and discussion

**Figure 2:**
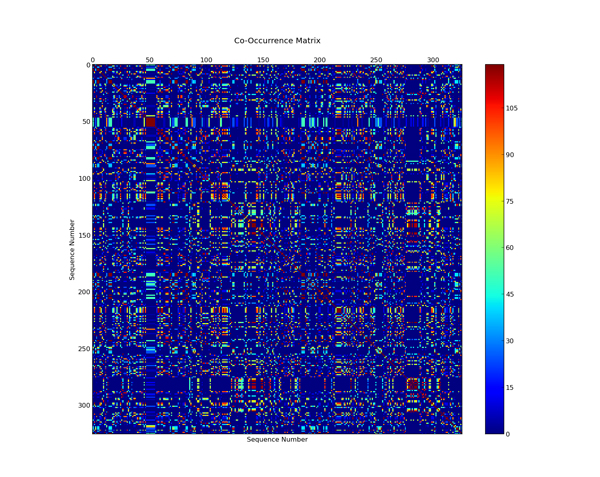
**Co-occurrence matrix for the 12 clusters**. The colors indicate the number of times given sequences in the data set occur in the same cluster. Red values represent high co-occurrence and blue low co-occurrence, both of which indicate a low amount of variation between consecutive runs of the clustering program.

We have studied several SOM architectures to see the effect of clustering of the Laccases with many descriptors. The clustering algorithm was run 500 times for each SOM map size. The clustering performance of each descriptor is listed in Table 3. Physicochemical properties showed the best average performance among all the feature vectors providing 12 clusters as optimum cluster size. This is also in close agreement with the study for feruloyl esterases classification where the strongest descriptor was the composite vector combining amino acid composition and physicochemical properties [17]. We also performed a co-occurrence matrix analysis to see the consistency of cluster instances in each group. The physicochemical property descriptor shows consistency in cluster instances between runs and different SOM dimensions. The co-occurrence matrix is shown in Figure 2. The 6x6 SOM dimension gave the best run with a DBI of 0.37 with an inter-cluster variance of 0.0088 and intra-cluster variance of 0.0015. The performance of the physicochemical descriptor in each SOM dimensions is listed in Table 4. The proteins classified in each group after the clustering approach are listed in Table 5.

**Table 3:** Performance of different descriptors in clustering of Laccases using various SOM dimensions

SOM Dimensions	AAC	C of CTD	CT	DIPEP	MA	MBA	Physico-Chemical Properties	Sequence Order Coupling
**5x5**	13	11	12	5	8	13	12	18
**5x6**	13	15	5	5	4	15	13	22
**6x5**	12	14	8	5	5	16	12	24
**6x6**	13	15	11	6	5	18	13	16
**7x7**	9	17	7	7	6	22	11	39
**8x8**	11	13	9	9	11	14	14	45
**CDVD**	8.2E-5	2.5E-4	-0.13	-1.1E-4	-2.9E-2	4.7E-3	6.1E-3	-1.3E-4
**DBI**	0.89	0.48	0.95	0.61	0.62	0.42	0.37	0.12

SOM: Self Organizing Map, AAC: Amino Acid Composition, DIPEP: Dipeptide Composition, CT: Conjoint Triad, C of CTD: Composition of CTD, MA: Moran Autocorrelation, MBA: Moreau-Broto Autocorrelation, CDVD: intercluster - intracluster variance, DBI: Davies-Bouldin Index.

**Table 4:** The average clustering performance at each SOM dimensions for physicochemical properties.

SOM Dimensions	Intercluster-Intracluster Variance	Davies-Bouldin Index
5 × 5	0.005325395	0.385778113
5 × 6	0.006355107	0.365723878
6 × 5	0.006241709	0.363347417
6 × 6	0.0061449	0.372815039
7 × 7	0.006477438	0.332075399
8 × 8	0.006438212	0.3249138

**Table 5:** Distribution of Laccases in different identified clusters under each taxa.

Cluster Number	Bacteria	Fungi	Metazoa	Plants	Total	UniProt Accessions
cluster-0	1	23	2	5	31	Q12541 P17489 Q70KY3 Q12542 Q941X2 Q09HV5 Q8X1W2 B5MAF4 Q4VY49 Q68LM0 R9WWK4 R9WS76 I0AVQ6 Q7Z8S4 Q6E124 Q2VT19 G8A542 G8A560 G8A555 I3PL63 G8A529 T1UMR7 D2KZ04 D2KZ05 D0E8H8 S5Q958 Q38757 Q8W0V5 Q2PAJ1 G8XQW0 E1ACR6

cluster-1	1	34	0	5	40	Q0DHL2 Q339K6 Q8RYM9 Q12718 P33644 Q9P8G4 B6V331 Q6R5P8 Q6RYA5 O60199 G0WM60 R4JRR8 D3K4I1 Q5IR80 Q50JG5 B8Y3J5 Q96UK8 F2VPT7 C1JCL7 Q9P8B9 C0JRG9 C0JRG8 G4XIH4 Q7Z8S2 H9C4A2 D4AIA5 Q69FX1 Q69FW7 Q69FW8 Q1EPM3 G8A545 D2KZ06 D2KZ01 D2KZ07 Q716A3 Q716A2 Q308C0 B0JDP9 C0P5Q0 Q4VJ26

cluster-2	0	4	0	24	28	Q10ND7 Q8VZA1 Q0IQU1 P78722 Q5N9X2 Q02081 Q9SIY8 Q9LFD2 B3TLA6 C0JRG6 Q8S2A8 Q2PAJ0 Q2PAI9 Q9AUH9 Q9AUI3 Q9AUI5 Q9AUI0 B9HHK7 Q9FSC9 Q9ZQW3 K4P1L9 K4PCQ7 K4NZE7 K4P1P7 K4NZ22 B1PXG7 F4MKL7 M5AN30

cluster-3	0	0	0	8	8	O81081 Q9AUI2 O24041 O24044 O24043 O24042 Q9ZQW2 M5AP95

cluster-4	3	2	18	2	25	P56193 Q02075 Q8IV20 Q8BZT9 Q99US8 Q49I37 Q4U3X4 Q9VX11 Q8WPD1 M4GPQ6 Q8I8Y1 M9PLY3 A5YVV0 Q8I8Y2 U5EZN5 D5MRE2 D5MRE1 F6UMP1 U3CY61 U3E6Q9 K9IXR5 F8V189 F8V190 J9PBQ8 J9PBR2

cluster-5	0	66	0	1	67	O59896 Q02497 Q01679 Q12739 B8YQ97 Q9UVQ2 I1SB14 U3M7S8 Q1W6B1 D3YJ58 C9WKP8 O59944 G4XU43 Q8WZG3 A3F8Z8 Q9UVQ5 Q9Y780 B5MAF5 Q9UVY4 F4ZCH1 Q6H9H7 Q6UNT7 A8W7J6 Q6A1A1 O74171 O94222 G9M4T7 Q96UT7 Q68LM3 R9WUR2 Q68LM2 R9WS74 Q68LM4 Q68LM1 G9I8W6 Q68LM5 Q8WZI0 G9I8W7 Q8TG94 C0JRG7 C0JRG5 H9BT70 Q7Z8S3 Q7Z8S5 H9C4A3 Q9HDQ0 B2L9C1 Q96TR6 E7BLR0 Q2VT18 Q6STF0 Q69FX0 Q69FW9 Q6X938 M1GME7 C6G7V1 C1KDZ5 I1VE65 I1VE67 I1VE66 D2KZ00 D2KZ03 D2KZ02 D2KYZ9 Q716A0 B0JDP8 Q8W0V4

cluster-6	0	26	0	1	27	Q12729 Q96WM9 Q12717 Q8NID5 I3NL60 Q50JG4 Q50JG3 R9WT58 R9WU11 R9WWK7 I1W1V7 I1W1V8 Q7Z8S6 Q5I7J0 E7BLQ8 E7BLQ9 H8ZRU2 Q6RYA4 C5IXN8 Q2AAD1 Q6TH77 C1KDZ6 C1KDZ7 C1KDZ8 U5XIR0 B5G552 B2CMA7

cluster-7	0	23	0	4	27	Q12570 D0VWU3 Q2QUN2 Q5N7A3 Q5N7B4 Q9HG17 Q50JG6 R9WUQ4 Q8WZH9 R9WT55 C5NN27 C0JRG4 C0JRG3 C0JRG2 C0JRH0 H9BT71 D9J137 Q8TFL8 Q2HWK1 B5G554 B5G556 Q716A1 K9R5B2 E1U754 E1U755 A6N8N5 K4NZF4

cluster-8	0	4	29	10	43	Q9LYQ2 Q84J37 Q53LU4 Q2R0L2 P06811 Q2R0L0 Q2QZ80 Q9LFD1 F6N9E7 R9WU16 G8A520 Q7JQF6 A1Z6F6 Q8I8Y0 A7XQR9 B4F7L6 B5BR55 D4AH59 Q58IU3 Q58IU2 I0IV86 E9RH10 E9RH11 Q49I41 Q49I40 A7XQS2 B5B2D0 D5MRR9 D5MRE3 D5MRS1 D0E8H2 D0E8H7 D0E8H4 D0E8H5 D0E8H0 D0E8H6 D0E8H3 D0E8H1 U6A581 Q4ZGM4 C9E6Q3 Q6TDS6 B2M153

cluster-9	0	1	0	11	12	Q9FY79 Q9LMS3 Q02079 Q9ZPY2 Q5N7B3 G0WXI9 G0WXI5 Q8L4Y3 Q9ZP47 Q2LD62 B9HHV7 G8Z904
cluster-10	1	1	0	4	6	Q9SR40 Q6STE9 Q8W0V6 Q2PAJ2 P93366 Q72HW2

cluster-11	0	0	0	15	12	Q6ID18 Q9FLB5 Q1PDH6 Q9FJD5 Q56YT0 O80434 Q9AUI1 Q9AUI6 Q9AUI4 I3W7E6 K4PCR3 K4P1M3

**Figure 3:**
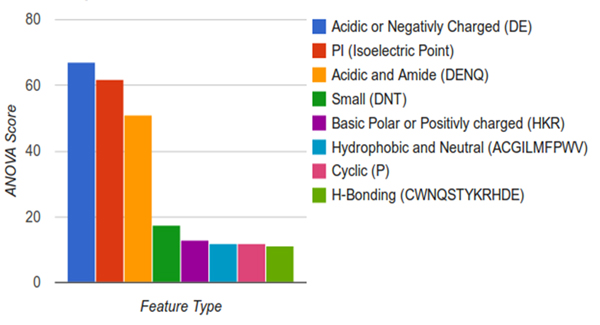
**The best physicochemical properties between all classes using ANOVA feature selection**. Larger numbers indicate stronger correlations between classes. Scores for other descriptors are not shown as they have ANOVA scores near zero.

**Figure 4:**
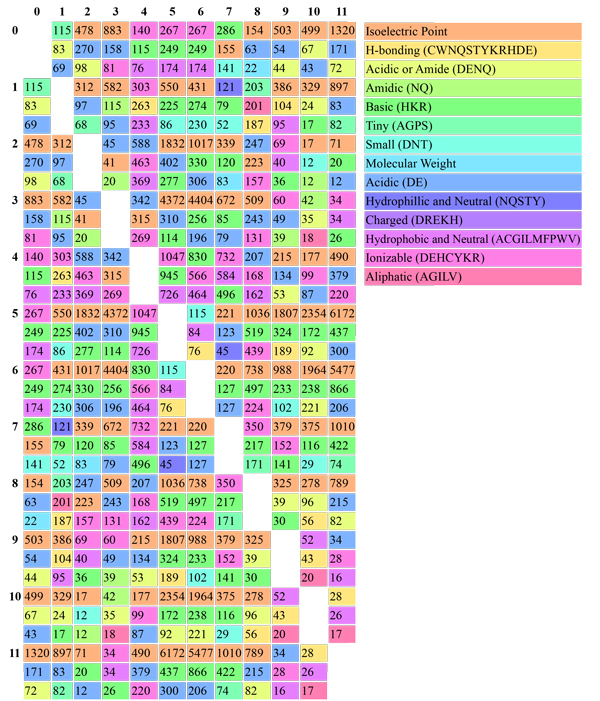
**Distribution of best three physicochemical properties between each class for the classes generated by LacSubPred**. The numbers represent the ANOVA score. Larger numbers indicate stronger correlations between classes. This indicates the best features and extent to their contribution for the distinction between the classes.

Analysis of the taxa in each class revealed that the majority of the classes were dominated by single taxa as reported in Table 5. Several review papers containing large tables of experimentally validated Laccases with various properties were considered to validate the clusters. Unfortunately, these were difficult to draw patterns from as the substrates tested varied widely and heterologously expressed Laccases often have drastically different activities due to different amounts of glycosylation [15,40,41]. To better understand what is driving the distinction of different classes, feature scaling was applied to the physicochemical properties of all classes together, as well as each class against each other. The major contributing features were the percentage of negatively charged amino acids, isoelectric point, and the percentage of acidic or amidic groups. The detailed information about the significant features is shown in Figure 3. This is particularly interesting as Laccases as a group operate over a wide range of pHs while individual enzymes seem to have fairly specific or broad pH and substrate ranges [41]. Also, it has been reported that different Laccases produced by the fungi *Coriolus versicolor *were easily distinguishable by their isoelectric points [42]. The differences between classes in terms of physicochemical properties, the best features were calculated for all classes and shown in Figure 4. This showed that the variation seems to be strongly influenced by acid/base properties, and next to the small residues or aliphatic residues. The isoelectric point occurred most often within the top three features with 45 cases, followed by basic amino acids with 34 cases, acidic with 32 cases, ionizable amino acids with 23 cases, acidic and amidic with 13 cases, charged residues with 12 cases, *h*-bonding and small amino acids both had 8 cases, tiny with 6, neutral and hydrophobic with 4, aliphatic with 4, hydrophilic with 2, and molecular weight with 2.

Additionally, we analyzed the descriptor values for physicochemical properties and amino acid composition between classes with a standard *t*-test. The *t*-test results of the AAC features between the 12 classes are listed in Additional file 2. It shows that Ala, Cys, Asp, Glu, His, Lys, Met, Asn, Arg, Ser, and Thr vary significantly between the classes. This is particularly interesting as the amino acids which have the highest amounts of statistically significant differences between classes seem to be involved in important aspects of Laccases. For example, the top two amino acids are aspartic acid and lysine with significant differences among 51 of the 66 possible class comparisons. Aspartic acid plays an important role in many Laccase catalytic domains such as: assisting in substrate channels in basidiomycete Laccases, affecting Laccase activity of C-terminal domains when mutated in bacterial Laccases, and assisting in the exit of protons from the N-terminal domains of bacterial Laccases [43-45]. Lysine can also be found widely in catalytic domains, for example C-terminal lysines have been implicated in the inactivation of heterologously produced Laccases [46]. Aside from function, lysines are also widely used as a cross linking target to bind Laccases to various materials [47-49]. Glutamic acid had the next most significant differences between classes. This was observed in Leu-Glu-Ala motifs which follow the copper ligating histidines and are thought to be related to Laccases with higher redox potentials [50]. Further, Asparagine closely followed with 41 significant differences. Many Laccases are known to contain asparagines which serve as sites for N-linked glycosylation [51]. These sites have been shown to be involved in regulation of Laccase activity through catalytic sites such as the Leu-Met-Asn motif which often replaces the previously mentioned Leu-Glu-Ala motif [50]. N-Glycosylation has also been found to provide protection against proteolysis [51,52]. Other types of glycosylation such as O-linked glycosylation are also major factors, so it comes as no surprise that both serine and threonine are high on the list [52].

**Figure 5:**
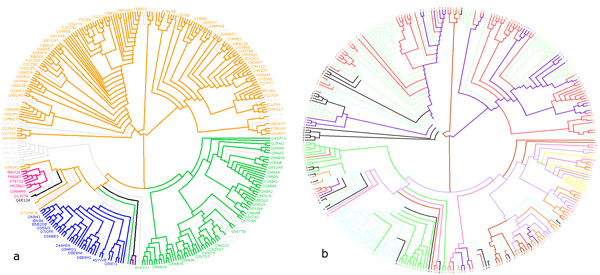
**Figure 5a. Phylogenetic tree from a multiple sequence alignment using the high quality Laccase dataset form the used for clustering**. Different colors represent different classes from the Laccase Engineering Database (orange: basidiomycete Laccases, pink: ascomycete-Like MCO, blue: insect Laccase, purple: Fungal Pigment MCO, black: fungal ferroxidase, yellow: bacterial CueO, green: plant Laccase). **Figure 5b**. Mapping of Laccase groups predicted by our clustering approach in the phylogenetic tree derived from the data used for clustering. The colors represent the 12 classes and also numbered as predicted by our approach.

In our other statistical analysis, the *t*-test results of the important physicochemical properties as identified in Figure 3 are listed in Additional file 3. It shows that all the physicochemical properties identified to be important in discriminating between classes are also significant. We believe since the generated classes contain many significant differences in physicochemical properties and the amino acids with high numbers of significant differences also strongly related to Laccase function, these classes may indeed represent different functional classes of Laccases. To investigate the classes further, a cladogram was constructed from a multiple sequence alignment using the sequences used for clustering. We then mapped our clusters and the classes from LccED to the cladogram Figures 5a and 5b respectively [15,16]. Despite many of the clusters being dominated by a single taxa, when mapped to the cladogram they are widely dispersed throughout the taxonomic regions of the cladogram. This contrasts sharply with the LccED classes which largely only follow taxonomy. Many of the neighbors in the tree are composed of enzymes from the same or similar organisms; these could indicate Laccases of different function from within an organism.

### Classification framework

To allow for the classification of newly discovered Laccases and Laccases with no experimental evidence, a Support Vector Machine-based classification system was developed. To accomplish this, 90% of the Laccase data collected was used for 5-fold cross-validation and the remaining 10% kept aside for independent testing. As physicochemical descriptors were used to build the classes, physicochemical properties were also used to develop the SVM classifiers. The developed models were further used to classify sequences annotated as Laccases with "homology" or "predicted" level evidence in the UniprotKB database.

### 5-fold cross-validation

The performance of the classifier in 5-fold cross-validation for all classes is reported in Table 6. The results show that the model achieves the overall accuracy of 99.03%, MCC of 0.9367, precision of 94.20%, sensitivity of 94.20% and specificity of 99.47%. The overall specificity is extremely high indicating a low rate of misclassified sequences. Considering the classes individually, the highest metrics achieved were MCC 1.0 and accuracy, specificity, and sensitivity of 100%. The lowest performance was accuracy of 98.98%, MCC of 0.7252, sensitivity of 80% and specificity of 99.31%.

**Table 6:** Performance of physicochemical descriptor classifier in a 5-fold cross-validation test.

Cluster #	ACC (%)	ERR (%)	MCC	PER (%)	SEN (%)	SPE (%)	FN	FP	TP	TN
cluster-0	98.63	0.35	0.91918	96.15	89.29	99.62	3	1	25	264
cluster-1	98.98	0.69	0.72522	66.67	80	99.31	1	2	4	286
cluster-2	98.98	0.01	0.8483	100	72.73	100	3	0	8	282
cluster-3	98.63	0.35	0.9356	97.06	91.67	99.61	3	1	33	256
cluster-4	98.98	1.02	0.93961	89.29	100	98.88	0	3	25	265
cluster-5	100	0	1	100	100	100	0	0	7	286
cluster-6	100	0	1	100	100	100	0	0	23	270
cluster-7	98.98	0.69	0.96881	96.72	98.33	99.14	1	2	59	231
cluster-8	99.66	0.34	0.97797	96	100	99.63	0	1	24	268
cluster-9	98.98	0.35	0.93086	95.65	91.67	99.63	2	1	22	268
cluster-10	100	0	1	100	100	100	0	0	39	254
cluster-11	99.32	0.01	0.90134	100	81.82	100	2	0	9	282

**Overall**	**99.03**	**0.01**	**0.9367**	**94.20**	**94.20**	**99.47**	**-**	**-**	**-**	**-**

ACC: accuracy, ERR: error, MCC: Matthews Correlation Coefficient, PER: Precision, SEN: Sensitivity, SPE: Specificity, FN: False Negatives, FP: False Positives, TP: True Positives, TN: True Negatives.

### Independent testing

Performance results on an independent test data are listed in Table 7. The model also provides higher performance with an overall accuracy of 97.98%, error rate of 1.02%, MCC 0.8678, precision of 87.88%, sensitivity of 87.88% and specificity of 98.90%. It should be noted that the MCC of cluster-3 was zero. However, this class contains only one sequence and performs well in cross validation, so we believe it is still credible.

**Table 7:** Performance of physicochemical descriptor classifier on an independent test data.

Cluster #	ACC	ERR	MCC	PER	SEN	SPE	FN	FP	TP	TN
cluster-0	100	0	1	100	100	100	0	0	3	30
cluster-1	97	0.03	0.851	100	75	100	1	0	3	29
cluster-2	100	0	1	100	100	100	0	0	3	30
cluster-3	97	0.03	0	0	0	100	1	0	0	32
cluster-4	100	0	1	100	100	100	0	0	2	31
cluster-5	97	0.03	0.909	100	86	100	1	0	6	26
cluster-6	100	0	1	100	100	100	0	0	3	30
cluster-7	100	0	1	100	100	100	0	0	3	30
cluster-8	97	0.03	0.851	100	75	100	1	0	3	29
cluster-9	100	0	1	100	100	100	0	0	1	32
cluster-10	97	3.03	0.696	50	100	97	0	1	1	31
cluster-11	97	3.03	0.696	50	100	97	0	1	1	31

**Overall**	**97.98**	**1.02**	**0.8678**	**87.88**	**87.88**	**98.90**	**-**	**-**	**-**	**-**

ACC: accuracy, ERR: error, MCC: Matthews Correlation Coefficient, PER: Precision, SEN: Sensitivity, SPE: Specificity, FN: False Negatives, FP: False Positives, TP: True Positives, TN: True Negatives.

### Confusion matrix

Confusion matrixes were made in order to better understand which classes are more similar to one another. The confusion matrix for the independent test set is shown in Table 8. According to the confusion matrix, it appears that few proteins in classes 1, 2, 8, 10 and 11 are predicted as other classes. The results in confusion matrix show the efficiency of the developed classifier in predicting the samples correctly.

**Table 8:** Confusion matrix for the predicted Laccase subtypes from 5-fold cross-validation testing.

		Predicted class											
	**clusters**	**cl-0**	**cl-1**	**cl-2**	**cl-3**	**cl-4**	**cl-5**	**cl-6**	**cl-7**	**cl-8**	**cl-9**	**cl-10**	**cl-11**

**True Class**	**cl-0 **(28)	25	0	3	0	0	0	0	0	0	0	0	0
	**cl-1 **(36)	1	33	0	0	0	1	0	0	1	0	0	0
	**cl-2 **(25)	0	0	25	0	0	0	0	0	0	0	0	0
	**cl-3 **(7)	0	0	0	7	0	0	0	0	0	0	0	0
	**cl-4 **(23)	0	0	0	0	23	0	0	0	0	0	0	0
	**cl-5 **(60)	0	0	0	0	0	58	1	1	0	0	0	0
	**cl-6 **(24)	0	0	0	0	0	0	24	0	0	0	0	0
	**cl-7 **(24)	0	1	2	0	0	0	0	21	0	0	0	0
	**cl-8 **(39)	0	0	0	0	0	0	0	0	39	0	0	0
	**cl-9 **(11)	0	1	0	0	0	0	0	0	0	9	1	0
	**cl-10 **(5)	0	0	0	0	0	0	0	0	1	0	4	0
	**cl-11 **(11)	0	0	1	0	0	0	0	0	1	0	1	8

values in parentheses represent total number of sequences present in each subtype.

### ROC curves

**Figure 6:**
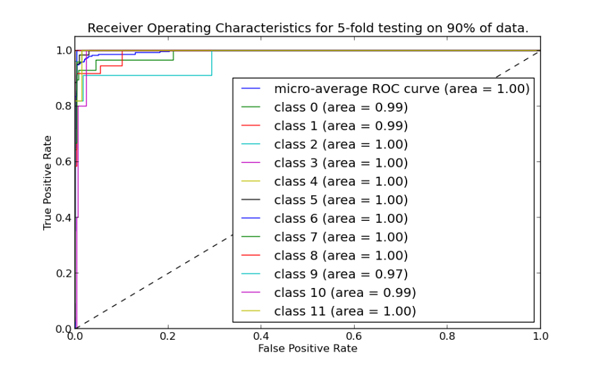
**ROC curves of different classes in a 5-fold cross-validation test**. Area under curve for each enzyme subtype is also depicted.

**Figure 7:**
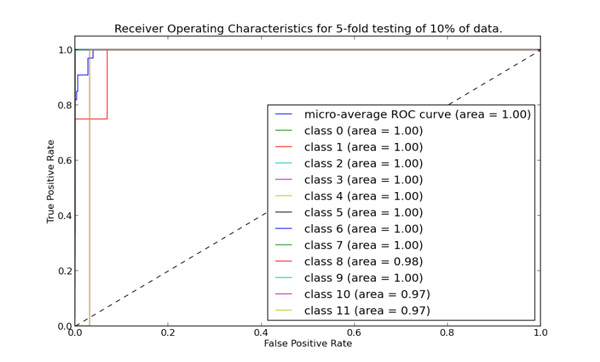
**ROC curves of different classes generated from the best model (physiochemical model) in an independent test**. Area under curve for each enzyme subtype is also depicted.

ROC curves are important to consider for prediction systems to give an accurate measure of credibility and or reliability. Each point on the curve is based on the confidence score thresholds of a single classifier. Each ROC curves compute the area under the curve (AUC). This indicates the probability of positive sequence having a higher value than a negative sequence when two are selected at random [53]. The more shift of the curve toward left, the more accurate the predictor. We calculated the ROC curves for each class for 5-fold cross-validation and independent set testing separately. The ROC curve for 5-fold cross-validation is shown in Figure 6 and for independent set in Figure 7. Each contains a line for each class in the prediction system as well as a line showing the average performance of all classes. All classes show excellent performance with lines very close to the left side of the chart, indicating a high rate of correct predictions from these models. Indeed, the overall area under the curve rounds up to 1.00 showing the reliability of our classifier.

### Functional annotation of different classes with domain maps

To investigate the role of domains in the functional variation between different classes, we generated domains maps for the sequences in each class. Eleven different types of domains were found to exist within the dataset. The frequently occurring domains are PF07732, PF00394, PF07731 and PF02578. The first three are mostly found in plants and fungi and the domain PF02578 found mostly in bacterial or mammalian origins. Class 4 contained a couple of polyphenol oxidase domains and tyrosinase domains. The domain maps generated for all the classes are shown in Figure S1 in the supplementary material. The majority of the domain maps were highly similar within and between classes with respect to domains present. However, there were some differences between the positions of the domains. We believe that these differences in the relationships between the positions of the domains could also account for functional differences.

### Classification of Laccase homologs from UniProtKB

The efficiency of our prediction approach is tested by identifying the Laccases in UniprotKB with homology or predicted level evidence. Out of the 1656 sequences retrieved, 1587 were predicted to one of the 12 classes and reported in Table 9. These annotations could be a good resource to the scientific community working in these areas.

**Table 9:** Classification of UniProt sequences with our method for those annotated as Laccases with homology or predicted level evidence in UniProt KB database.

Cluster-0	190	Q7XE50 Q6Z8L2 P74606 G4IJ94 V5XWB3 B2C6F1 Q08AC3 Q5BEX9 J7MF98 C0ND57 B2WK27 C0NNG0 B2VRC3 M5BZA6 R1G4L9 D6BQM4 D6BR75 I8U4Z0 F8Q484 F8Q476 K9MGI8 K9MEL4 K9MEY0 K9MEY3 A1YJE8 C5G0U9 F2RR98 C6H349 H1V814 C6HJZ6 B8P5X0 D7F612 H6BR26 M7SF91 R7SPG3 R7SPI3 L7JI99 L7JBV3 L7IRC9 A2QS62 A2QL29 A2QL49 G2WRK6 G4ND85 G4MZV7 L7IFQ2 L7IP33 L7I255 K1X050 I3VB24 G7X777 N4U815 N4TWZ3 G3XU06 C5GE22 N4TVC5 G3Y787 Q6VMB7 Q6VMC0 K9HCS9 K9HQU3 K9I712 K9H938 K9I022 K9I2L1 K9HG94 K9HJU4 K9HNK5 E4ZMJ1 E5AE29 D3GBU0 D2D2A3 B8QJ10 D2DX16 E9R598 M7UAA8 V5I3G1 F0U6R9 F0UJJ0 F8P301 E9DUY5 E9FCN3 E9F648 E9F7W6 E9F671 E9FDX0 M7WUL3 V5XX03 V5XVN3 V5XWB7 R7SEA0 N4VBQ5 N4VMC7 B8NWQ2 B8MZ52 B8NPM5 B8NMX2 B8NBJ1 S0DUR9 S0E0U5 R8BVQ5 F2T3A3 Q7S2V2 Q7RYF6 Q7S6W1 D8QAX5 Q6E0Y2 Q96VT5 Q9C497 C0SBX8 B2DFU1 A8NCW1 Q08AC0 A8NCV5 A8N4C5 Q08AB1 Q08AB0 G3JB06 H0EYN9 Q96TR4 N1RLU0 N1RPZ1 B8MHG8 Q872X3 Q8TFE3 V2X2F4 V2X113 V2XZ31 V2WZW0 V2XKE2 V2XJ10 V2WNA2 V2X2H5 V2WZ01 C5JSC6 A1YJE9 M2QJC6 A7EIG9 M2R2P1 L2GG91 L2FPD4 R9A8V6 F4RHU0 F4RB81 F4R7H8 F4R9G7 Q8TFD9 C3SAH7 J9W0J5 Q5KEA0 J9VY90 Q5K7H5 S7PZA1 G2Q560 G2QFD0 S7RMW6 G2QG31 M1W227 K1PE03 K1QYL2 K1PU89 F2UPC3 K7TYL9 K7U2C8 M8AXH2 M8BNT5 B6B2Y8 K7SIF5 S3J3R5 B6RA59 F6L7B5 L7P6C5 L8NIT9 L7E7D5 G6FWW3 F5ANG7 G8IJH7 Q8GB87 L8PIS4 Q8NLH5 Q3ICN9 K9PVA8 F4KPJ9 H2G3K4 K9V2J8 G8PGI1 H2HE99 H2GRM9 M9X7P2 J9GZX3
**Cluster-1**	120	Q0DHL5 Q69L99 O66554 Q87AR8 P67256 P67257 P33663 Q9PET8 P45496 M5CF06 M5C3X0 L7JBX6 B7UB78 L7IH98 R9PCC0 E9ELE3 S4PNX6 M7YVZ5 M8AE68 M7YSX1 M7YW17 M7ZS72 M8A139 G7KE42 G7JUW8 G7JZP3 G7JMZ0 K7UTI2 K7TRU9 M8BZ91 M8ALT2 R7W1V7 N1QW01 M8BGG3 M8C3K6 M8B5Q9 N1R3U3 B6REK9 G7ZJ86 B9IG57 B9HCK0 B9HCJ9 B9GMJ0 Q337U9 V6ZGQ2 D2KMW3 I7AL37 Q4H436 V8K3V9 V8HET4 G6HM36 E9YFX5 V2VMN4 S7P3Q8 V6ZS55 T2LUJ3 F9U2L8 V7J4U2 U1T5B7 V5PQZ3 I4JMD8 V5AFY1 R8ZME9 M5SSI1 F7NAJ8 S0H0G8 V8L0G5 T0B7N3 V7K0E4 V8CWG5 M6WH33 T5KM43 U2AIM6 G0PZI6 V7KQ45 S7TXN1 F5ANG6 V7L5L4 T0Q5D9 M3VA17 N9VZ85 K0XMX1 K8EJK7 M2VPI6 D6AWT6 U4M0A2 U5RLM8 R9ZJN7 U5R119 L0DYT4 S5F6G0 T1XAQ0 F0Q362 V6K2N9 T2EB93 T2E701 H6MRY5 T2ECM1 T2EN58 E8U3Z4 G2SEA5 U5WK13 G7ZEA1 F4GD53 G0GDH2 F6AU05 G0K0P6 F6B3B1 U3QKS9 G0JKU1 F6DH61 K4LEN3 F4CL81 S5NVM5 V5XFV5 F0RPC2 F8AYB4 F2NPR3 T0YGH7 T1BRU0

**Cluster-2**	7	G7L1B5 G7J2E0 G7J2E1 B9IG57 B9I2G5 B9HCK0 B9HCJ9

**Cluster-3**	349	Q99046 Q0JHP8 Q87AR8 Q9RT03 P67256 P94338 P67257 P33663 Q9CCE3 Q9PET8 P45496 Q08AB4 Q08AB6 Q08AB3 Q08AC4 Q9Y781 C1GYT7 M5CF81 M5BXM9 J0WTP5 H1VYH8 D3KDK0 I6QS85 Q6VMC1 Q9Y782 Q875H1 B0DD17 B0DD16 G4XU44 E9FDT4 A1D1J3 D8QAX5 C1G7M9 C5HL40 Q0KHD1 Q08AC5 Q08AB5 Q08AB8 A8N889 A8N4M0 A8N893 H0EYN9 U5QB88 U5QEW4 U5QB94 F0XLH6 V2X2V9 V2WLL0 V2XHV1 V2WNA2 V2WKD7 O13420 M7YWY1 M8AE68 M7YE76 M7YDG7 M8A1Z6 K7UNU7 K7U8Y5 K7TU80 K7UAF3 K7TYL9 M8BD16 M8C508 R7W4T4 R7VZA0 M8BNT5 V4Q585 V6ZGQ2 S6WSA8 U6ZZ13 V8K3V9 S6P2D4 E9VBD2 I3AGZ9 C4HZR3 S6W0B1 S7VA95 S6KUV8 V2K349 V6VBS8 S6T1H2 L7HFV6 V8HET4 J0YDS8 E9WV02 S6S577 U5VK00 U7P8G6 F4QUH2 E9Y3Z4 V5P9V4 V2VMN4 S7P3Q8 V6ZS55 S6TNM2 S6MJF0 K2J3S4 C4HCI8 U3HA11 V8D9D3 T2LUJ3 T5FBY6 U2UAL5 R5B3Q9 K5BB49 D2SYY6 F9U2L8 S6N9C1 U7HPI8 F3X0N9 S0ACK2 S0AGR8 V4H7V8 V7J4U2 U1T5B7 S6UPW2 S6PLN6 V5PQZ3 N4VVL2 I4JMD8 S6RKW7 K4YE07 V5AFY1 L9U8Z5 M5QZ37 L7GS59 S2KKX0 U7NUT4 E9VXJ8 S6PW15 S3MSW6 R5IKW2 T5GFQ3 T5FTT4 S3FWN6 S7TK32 U0E669 S5RYB0 U2WG75 N0D6V4 S6L531 G3IUL2 S3E4U6 L8Y324 M5SSI1 L7FVM2 V8EKS3 D4X8B0 S9S1I1 H1KST3 S3F1H1 F7NAJ8 S0H0G8 G2DYC3 V8L0G5 D5FL31 T0B7N3 I4N140 I8NS20 V5CG64 M7WBY4 T5HP32 U3HXE8 E9X8B9 S6V7D6 B0FMT2 T5EBW6 V4WZB0 U7NNM4 V7K0E4 T5IE49 T5FRA3 I7RQ32 S2KX53 V8EM40 B5GZZ8 T5LC46 A3QVH2 S6REG0 U1FBQ2 M7VYD7 F4QSG8 H4FDU8 K6AF68 K6CUJ1 V8CWG5 E9XI50 M7VVH4 R7HNS6 N9U861 T5KM43 T5F756 V5PFI4 U2AIM6 E9ZDU1 T0IJI6 K5YI60 G0PZI6 V6JAD1 V4V0B4 V7KQ45 V8QMC0 R0FHH1 T5IKC4 V8G7M9 M7RCK7 I7IET5 E3VW04 R4YX27 V7DIM5 M3AK06 U1UTN2 G8XUN7 U5AUF6 G4E2X0 S7UGQ3 R6T8W7 G6E0Y0 S6JXK8 I7PQJ2 I7AYT4 V2WWR6 G4I2W0 L7FPM1 S3F3H5 S3EKB6 E9VNA5 M3VA17 C4H9Z9 S6MLH9 I8MF47 V8R2K2 M7UZV1 N9VZ85 V5BX08 K0XMX1 H0G5H7 A3ZAS6 S6N0Z2 I7QN70 J7SN90 G4FII2 J9S5G3 M2VPI6 U7RH62 E9Z1L2 E9WJR1 V4MW22 E0IYQ8 Q1CLW9 C9LW01 U4M0A2 L8N5P4 F2K1G6 L7ZQF4 G8TX43 R9ZJN7 B7N7Z3 I6XY59 G9A3R6 I4CP94 C4ZRL6 M9R493 U5LY75 G0DJC3 M4JYB4 I6ZBU4 F6AGJ9 L0DYT4 F6CIV1 H2HZF6 B1VQH9 F8F070 S5F6G0 G0A3Z5 T1XAQ0 I3XBF5 F0Q362 F6EXD3 T2EB93 H6MRY5 T2EN58 B7M164 G8S901 M9S5N5 E8U3Z4 M4WRW0 G2SEA5 C6UM84 F8GKM0 G8AZV3 G0AYB6 B1XC98 B7NI83 U5WK13 G7Z7B9 L0FEJ1 G7ZEA1 I0AC26 G0C4M5 F4GD53 G0BZP7 F6AU05 G0K0P6 J7L6N5 S6A106 F6B3B1 M9RKU1 B7LFZ0 D7ALZ3 U3QKS9 F6DXN2 B7LVY7 F3Z0Y9 T2MUS2 V5UKF6 G0JKU1 G2S6B7 F6BWK2 I4B8C3 F4LJ75 F4CL81 H2IFJ7 U7DKH5 A6BY94 V5T2H8 H1YL11 F7ZEF4 R9UYU4 A1A7G9 Q39Q28 F0RPC2 E4PNH4 C6UWM8 Q74H10 S5Y607 F8AYB4 M5DVF3 G0B840 G0B416 G4PUB6 G0BKW2 G0BPY6 F2NPR3 B5EBA0 B7MBA6 T0YGH7 T1AI46

**Cluster-4**	29	Q0DHL5 Q0IP28 Q5N9W4 J3P0A5 M5BZV9 F8MRY7 B0DUR0 G4UW76 G4UY21 Q7S1M8 V2WYW9 L8WE35 M7YD49 M8AA34 M7YW17 M7Z3H9 M7ZXZ0 G7KEX5 G7L1A9 K7V3L7 M8C2V0 M8BD16 M8BYM7 R7VZA0 G7ZJ86 Q9FSC9 B9HHK7 B9HC76 D7M768

**Cluster-5**	2	G7L3F3 G7JZP3

**Cluster-6**	511	Q7A617 Q8NX32 Q6GA25 Q6GHP8 Q5HGP4 Q9PN78 Q49WW9 P33664 Q4L5N8 Q9ZD53 Q5HQ05 Q8CSX5 O31726 Q9ZHA4 V6DRF7 M5CFX1 M5CAM2 M5C720 M5C185 M5CGP5 M5C589 M5C753 A2QZQ7 K0K866 N4UAJ2 N4U7Z5 N4U1H6 Q4WQY8 G9BMJ0 E9E686 E9F2R7 B0Y585 S0E9D8 S0E4X8 S0E1Z4 S0EL60 E4V6P8 E4V687 U4LLJ8 N1SBH2 N1S1T2 N1RPA1 N1RS78 N1SBA6 V2WQU2 V2XKE2 L8WTK6 L8WJY6 F4RH14 Q8TFE1 M1WHU6 M1WAS8 K1Q401 K1R9J5 K1QCA2 K1Q8Q1 K1PMS4 K1RX04 K1QWK0 K1QE27 K1QDM4 K1QKD0 S4PM90 E1ZWT8 E1ZZD9 E2BAT2 E2B8J4 E2B9K7 F4WZP2 G6DFM6 R1EWW0 U6C8D7 G4U403 U3UB68 U3UB71 G4U404 G4U402 U3UB86 B3WFP2 V6QDX8 I7RCW2 T5HR61 T4H7F2 T4CHL1 T4RCH9 I7QE77 T3BR38 T3X6W0 L8N092 U6SRD9 V6Y5S7 T3LRV7 T2XUB5 I7WJ04 T3S4X4 I7R676 S4M6X3 E5VKZ1 V6QRD2 T3L2F0 T2WMR3 T0SSX7 T4QDE8 R5TX08 H8WGE4 I8HA90 T3P4N3 G6GI01 H8WGE5 T4NRV4 T3BGH0 T3MXD3 T3N9N4 I8F9W9 T3VTX7 T3HSI4 M7NA89 V4Q2M0 T4Z017 T2Y6R3 T4AC54 T4JL39 T5AW43 T3FJR6 H8WGE6 T2TE53 T3WQ59 S4LT46 I2HQT5 T4JIA9 K1LKK1 U4Z1T7 S7J0E5 T4SJK5 U1SVL4 T3M974 T3TC93 U4TFM5 V6YAR5 K4Z8D3 T3JCJ5 T4XU61 T2THC2 I6KRF8 V6X444 T3MQZ8 H8WGE2 T4DJW0 U1URF7 T4QEB3 H3UJI9 T4AVY0 T3SA96 D5MWS7 I7RQN7 T2Y3J2 I8HK20 T4VQU6 T2VWA2 T3U4V0 I7NMN7 I3RYX9 T4YBC3 T3SU48 S3FZ67 M1E4W8 E5VEH1 T4EYI7 T3YAB4 T4WEJ8 I8JLR4 R4YVC2 L8PW18 T3FDX0 U2G960 S4MJC4 T3CFI8 S9RGQ9 T4KB56 S4MUD5 I8R624 S7IVW3 H2CEH3 T3AKJ9 T3C4F9 V8G4P8 T4NJ03 T2TWX1 U7PCJ6 S9Z033 I7Z4V8 T3AMA3 T4GLA8 T4PHK4 V6QRF3 T3JQC7 T3Z9Z6 T3C1U6 R7LI33 T3GSF7 R6YU61 T3WGI6 S4LUD9 T3VEH2 T3D776 T3ZWS1 I6J5Z8 T4CLT6 T4I2Y2 V6XI18 T4KEL9 T4FBY8 E5V4S0 T4NKE8 T4HAP8 S4M8Y6 V8A3Q5 T4DD15 I8A9I5 U4YDV5 I7NP16 T3HCY2 T2YTP4 S5MW00 T4MM52 T4TYC3 T5LL74 I3I532 S4LFA3 I7U727 T3QSF5 I3IAJ6 I7RTJ4 T3LMQ3 U4X425 T3G5B3 T4W8X6 T4HAZ4 H8EFD2 T0CRL0 T2UFK7 T3VES0 T2VBQ4 U4X657 T4ALC0 T3XVF5 T4FIE9 V7Q487 T0ACN3 T2ZM17 T4PGA8 H8WGE1 I7VUN6 U5U549 R7A750 H8WGE7 T3DF94 G1E8V8 T3GST2 T4SHI2 T2ZH17 T0R613 I7VYZ3 T3KF92 R7C1G9 T3IGA6 I4XC66 V6X9T4 R7HW39 T4M8I0 T3XKW1 T4Y5N2 T2XHU3 T3Q5L5 I7MWS9 T4BN13 T4KVV0 I6JVS5 T3R8P5 E5VZ21 T4LTW2 T3Z9W4 T4KN38 T4UDD8 U2TN14 T3CSY2 R7JC65 U2ALG6 I6I5F4 T3RUH4 I8CF83 I7UBQ5 T4VCD0 I7S7M9 I6JYZ9 M7N823 T4UP53 T3F035 S4LPW8 T4Z8T3 A3QVH2 T4LEJ2 T3SX82 I8DF43 I7T5Y8 T3TN05 T3IG28 T4TK70 I7UQD4 T4HVY2 T2V0S5 T4X5M0 T3YD82 T4XHQ3 I8K1U9 T4RCM6 T4D1R6 T4TG37 T3UB07 V8Q506 T2VNC8 S4LZS5 U4XJ65 T4USJ1 T3K8P4 T4EF23 T4DZY7 J7FRV9 V6XQP2 T3V1E1 T2Z762 T4BBS0 T3GBZ7 T0AKR5 I7Z279 T3YY69 M5R626 T3AWT7 S7NEL4 M5RDU4 I8SCA2 V7ZZF7 T2QPY1 T0D1C8 R5Z7A9 T3XRL3 T3HPC0 S6JJK0 T4SVW1 T4FZI2 U6EKI0 T3P2U8 H8WGE9 T3EAL0 T2QKY1 U1VRD5 S4LAY4 T3A0K2 V6YE28 T3W8C7 F8NA39 T4C5U1 I7YR63 T4G7W0 I6IIJ1 T3PG48 M7XKG4 T0DFK8 T4S873 H8EJX8 I7S5R1 I6KQ20 T2WY85 V4T5T1 T2UPR1 S6IKA9 I7Q253 I7X2J1 V8PVE5 I8FEL5 T3J5E0 T4RUP8 T4Y0J7 S5DK30 S7K1S3 T4VUX5 I8BK88 T3NLY3 E5WXX9 T3L1J7 I8P8L5 T3UHH4 G4EZC6 T3K913 T4FKU8 T3QZJ3 B9W2C5 I7WSU8 U4YZ68 T4QHQ3 T2X3N3 T4WXI7 S6K0B0 I6HRL7 I7Z9E2 I7R5M8 T3DUH5 U4YKE5 T3M949 V6WZK8 T5AYI3 I7V8E8 T3PWT2 T4MUD1 T4LWE3 T4PUB2 M1LQ69 I6ZLM4 T4IMK7 T2R5W8 T0A454 I8U5C9 M1Z571 T2TN13 H8WGE8 T4ZA62 T4J0P9 V4TXP2 S4MAR9 T3R3Y4 R6A7E1 R6QFK7 I8HIH5 T4E5P6 I7T8U6 T3ENB4 S6F922 T3FBQ3 J9S5G3 H8WGE3 I7PV33 U4XYD8 T2WCS4 F6F5H9 F6FAN1 F6F865 L7VU04 K0LRJ8 F6FD51 F8EL41 G2PUB7 G2RJ98 F4E312 F6BKT6 F4EK15 E6RIC9 S5P4Q8 I0F413 Q3ICN9 I3VS24 F2NJT7 N0D000 G2SU96 M9T8B1 F2JS63 S5J299 B3PFW0 L8AB38 S4X6W3 U5L7H1 M9TBK0 M9T9N5 G2SHN8 E8VCU6 Q3A0C1 I6Z2M4 F2NUQ6 F0T009 F8CWY2 I2AZ79 I2AZ80 U5MNG8 G2TJW0 E0TU44 S6FHG0 F8E576 U5Y2D7 B7GFB8 N0D971 G2MRU7 F2LWH8 K4FQY0 I0U612 G0IFR5 D9RGB3 K0AYI8 S0A483 F0R1V1 G7MCN9 I4B8C3 C7ZWD5 E3E197 S6FK09 H1XUB2 K4MD70 D9RPK8 G0H8L2 F3ZT38 U5RXN4 J9GXT0 I7J6X7

**Cluster-7**	73	Q99044 Q08AB7 M5C1X8 Q5MBH7 F8Q492 K9MEY3 A1CNP4 Q6YA64 E0W6S1 B8PJH3 B8PB64 Q1AJM3 R7SP52 R7SWM9 O13448 Q8TFM1 D7F485 Q8X1W3 E1CGD5 Q9UVT6 C5HL42 Q9UVT5 Q6VMB9 Q6VMB8 Q69AX8 K9HJU4 I6V2C5 R7RXX6 Q2V0Z9 B0DC12 S4VMI6 B0DD15 V5G506 F8P308 Q5MBH2 Q5MBH3 Q5MBH1 Q5MBH6 S8FIE4 Q12571 D8QME3 Q96VA5 D7F484 H9LVX1 B2DFU1 Q08AC2 A8N4D0 Q08AC1 Q08AC6 A8N3P5 A8N842 S4VGX6 U5QBI8 U5QBJ2 U5QEW9 U5QBE0 U5QEN9 U5QBD5 U5QEV8 U5QEN2 V2YDW2 V2X2N8 V2X7U4 V2Y5W2 U3PH31 M2QK34 M2QJC0 O13422 Q5MP11 S4VGN3 S7Q2E4 M7Y5L1 R7W718

**Cluster-8**	95	Q03966 Q12719 Q99055 Q99056 B2WJB9 R1EUQ8 R1GA89 F8QG75 K9MFH9 K9MEL6 A1YJE8 M7SKK0 M7S5C0 H6BQP7 Q50H77 M7SF91 R7SVI6 L7IRR7 A2QS62 A2QW24 A2QL29 G2WZW4 G4N0V4 O13456 L7I0R6 K1Y862 E1CGD6 G7X777 K9MGJ2 N4U4G9 G3XU06 G3YCK7 Q6VMB6 K9HG94 K9HRW5 Q8TG93 M7TM62 M7UCN4 M7TD67 R9P950 V5G6H1 E9EF39 F8PB06 B1NEY0 G2XUR3 E9EKY1 G2YUE6 G2XZ63 C9SND1 Q5MBH0 A0ZXN9 N4UQV6 N4V5N9 N4VL55 S8ED94 S0E275 R8BAM6 Q7S6W1 Q7S4C0 A1YJF0 B6Q5M9 A8Y7S9 Q08AB9 A8N4I7 H0ET37 G3FGX5 H0EM62 N1RP79 B8MHG8 F0XG23 F0XJC5 Q3KRP1 V2Y238 V2X382 V2XTJ5 V2WXK7 V2X4B3 E7AIS4 A1YJE9 M2RE78 L2GCR5 L2FPB4 M2QYC3 M2QK38 R9AGK9 I2FSE5 E6ZSD5 B5G551 F4S552 F4RZ56 F4RZ43 F4R9G7 J9W0J5 Q5K7H5 S3BV84

**Cluster-9**	61	Q2RBK2 Q2QYS3 G5EGX4 J3PI40 B2VSS7 M5CEU4 R1GKT9 R1EPH7 K9FSV2 F8PY37 K9MFI2 K9MH70 Q50H78 R7T0U4 R7T2I7 L7J9Q6 A2QB61 L7IKL2 G7XIZ1 M7TSR5 M7UW45 B0CT29 B0DS50 B0DZT1 B0E060 F8NWN9 G2XZH4 G2Y1J7 Q5MBH3 K9GTR0 N1QF47 H0ET20 F0XK69 B5G553 Q8TFE2 B5G555 V2X3Y4 O13421 C5HL41 F4RZG5 S7PZA1 S7QCZ9 K1PTU8 M7YKQ5 M8AVV6 S1SMV4 S1RWH5 S1RU27 S1SIK3 T5FBY6 V7A306 T5EGQ4 T5GFQ3 T5FTT4 T5EBW6 T5IE49 T5FRA3 V7DNX4 T5F756 T5IM14 T5IKC4

**Cluster-10**	110	A2XCN6 A2Y9C5 Q5ZCW1 A2Y9C2 Q6Z8L2 J3NMB3 J3PEH0 J3P582 B2VVH6 C1GYT7 B2VX28 C1GNR8 M5CEU4 M5BXM9 M5C753 F2RX26 F2RR98 F2SL00 F2SMD3 D7F613 L7IQ72 L7J535 L7J2C5 L7JNP2 L7IQT5 G2WRK6 G4N3F5 G4N5T0 G4MYQ1 L7I6M2 L7IJ97 L7IL92 L7IKQ9 L7INY7 K1X050 G7XK56 N4U815 N4UI31 A9XCN9 K9I2L7 M7UW45 D4AUP2 E9FCN3 C9S5U1 N4V8A2 N4V0M8 S0ECY0 C5H3G0 S0E4X8 S0DPV4 Q7S1M8 D4DAZ3 E4V6T0 C1GIK6 C1GFF8 C1G7M9 C0SBX8 H0EWH4 N1S5Q4 Q8TFE3 Q8TFE4 Q8TFE2 V2X2V9 V2XU47 F2PUB0 F8WTI5 Q2HBW4 L2G1J4 L8WJY6 L8WN49 L8WY78 L8X1E3 F4RHU0 F4RNV7 F4R859 F4RZH0 F4R860 F4S2L7 F4RH14 F4RRN3 Q8TFE1 S3D280 K1Q1L0 K1Q8Q1 K1RX04 E2ANM3 E1ZWT9 E2AKS0 E2BNZ3 B0XBY5 B0WW28 B7YZT1 A1Z6F4 F4WZ78 G6D2R8 M7YWY1 M8A4C9 M8ALF7 M7YKQ5 G7K780 M8AS44 M8C210 M8BZH5 N1QX28 M8C8G7 D7LZX9 B5SBQ1 A3IX38 Q8XPV6 G7Z7N4

**Cluster-11**	40	O51423 Q92HU9 C5PJ66 L7JBX6 G2WR51 L7IH98 J3KH83 E9ELE3 L8WLA1 M1VV00 K1R9J5 K1PMS4 K1QP95 E1ZWT9 G7KE42 G7KBW6 G7ILB5 G7L9F5 G7J6C9 G3FF67 M8BJM6 N1R3Q4 N1QW01 N1R3U3 B9GF92 B9I2G5 B9I6L0 B9HHV7 D7M229 U4T622 S7J8T8 S7KUM4 T5KCD3 S7TXN1 F5ANG6 S7KHJ6 F0S0K4 F9YDA1 F4HEF0 F8C682

### Web tool for classification of Laccases

We have developed a web resource for the classification of the Laccase subtypes by implementing the machine learning models. It will be very useful to the researchers to characterize the newly found Laccase sequences. The tool can be found at http://lacsubpred.bioinfo.ucr.edu/. We have also provided the codes used to develop the clustering and classification approach as an open source package available at https://github.com/tweirick/SubClPred.

## Conclusion

In this work, we present a systematic computational approach to identify Laccase subtypes. First, a novel clustering method is developed to group the Laccase subtypes using the experimental data available in UniprotKB. Then a classification method is developed based on machine learning approach to generalize the functions of Laccases in each class. These identified groups can be a useful resource to the biologists to study the characterization of Laccases, particularly for researchers in the biofuel area.

## Availability

LacSubPred, the web resource developed form this study, is freely available at http://lacsubpred.bioinfo.ucr.edu/.

## List of abbreviations used

ROC, Receiver Operating Characteristic; MCC, Matthews Correlation Coefficient; SOM, Self-Organized Maps; SVM, Support Vector Machines; DBI, Davies-Bouldin Index; AAC, Amino Acid Composition; CT, Conjoint Triad; CTD, Composition-Transition-Distribution; DIPEP, Dipeptide Composition; MA, Moran Autocorrelation; MBA, Moreau-Broto Autocorrelation.

## Competing interests

The authors declare that they have no competing financial interests.

## Authors' contributions

TW collected the datasets related to Laccases from public repositories, wrote codes for clustering, developed algorithms and models, performed the calculations, figures and tables, and wrote the draft manuscript. SSS helped in model development, data analysis and tool building. RM helped in biological analysis and in editing the manuscript. RK conceived the study, participated in its design and coordination, and edited the final manuscript. All authors read and approved the final manuscript.

## Supplementary Material

Additional file 2 P-values designating the statistical significance of one cluster over the other based on amino acid composition differences; values calculated using the standard *t*-test. Click here for file

Additional file 3 P-values designating the statistical significance of one cluster over the other based on protein physicochemical property differences; values calculated using the standard *t*-test. Click here for file

Additional file 1 Domain maps for each of the Laccase subtypes cluster generated using *doMosaics *(http://www.domosaics.net/). Click here for file

## Declaration

Funding for the publication of this article has come from the 'start-up' funds provided to RK, account A01949-19900-44-CPK1, UCR.

This article has been published as part of *BMC Bioinformatics *Volume 15 Supplement 11, 2014: Proceedings of the 11th Annual MCBIOS Conference. The full contents of the supplement are available online at http://www.biomedcentral.com/bmcbioinformatics/supplements/15/S11.
