# Supplementary figures and images for: LacSubPred: predicting subtypes of Laccases, an important lignin metabolism-related enzyme class, using in silico approaches
Source: BMC Bioinformatics. 2014 Oct 21;15(Suppl 11):S15. doi: 10.1186/1471-2105-15-S11-S15 (PMC4251044; doi:10.1186/1471-2105-15-S11-S15)

Class-0


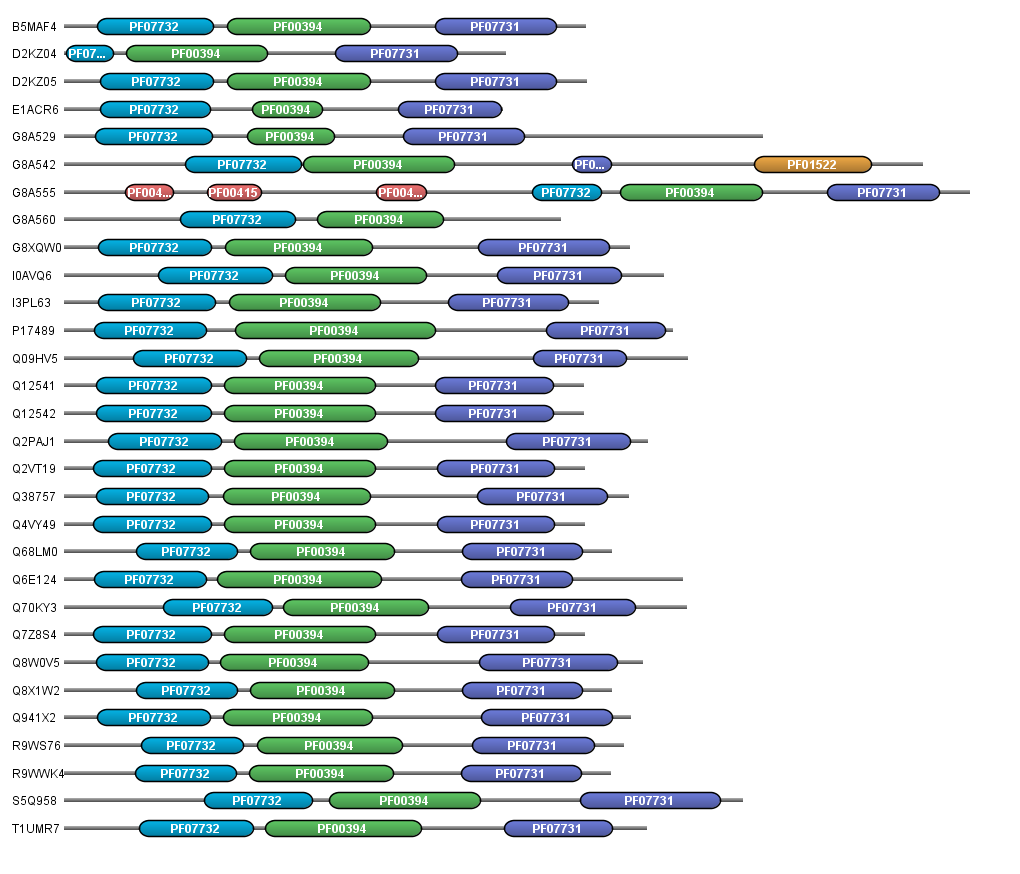


Class-1


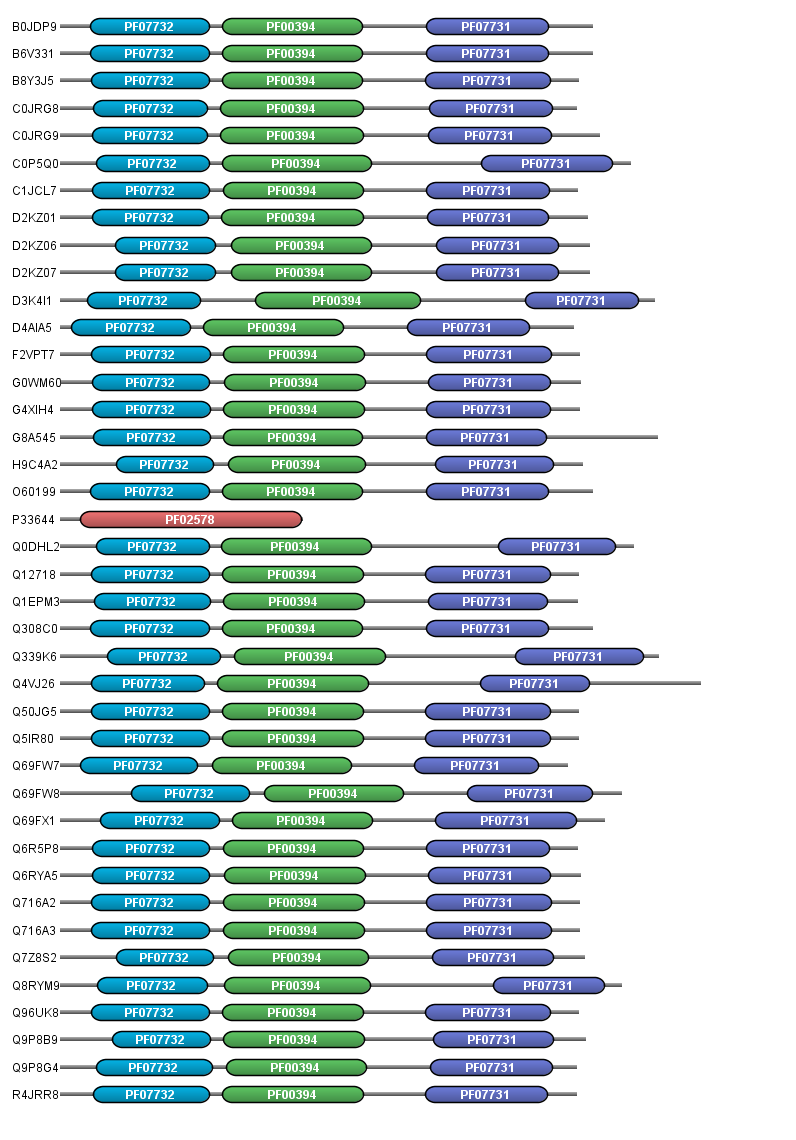


Class-2


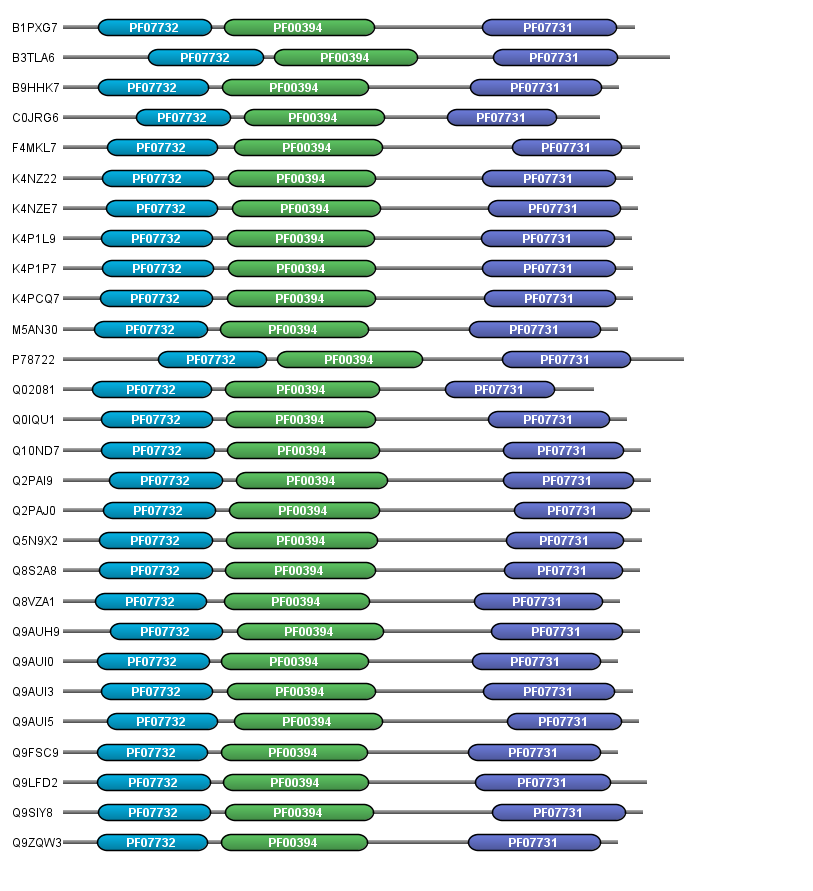


Class-3


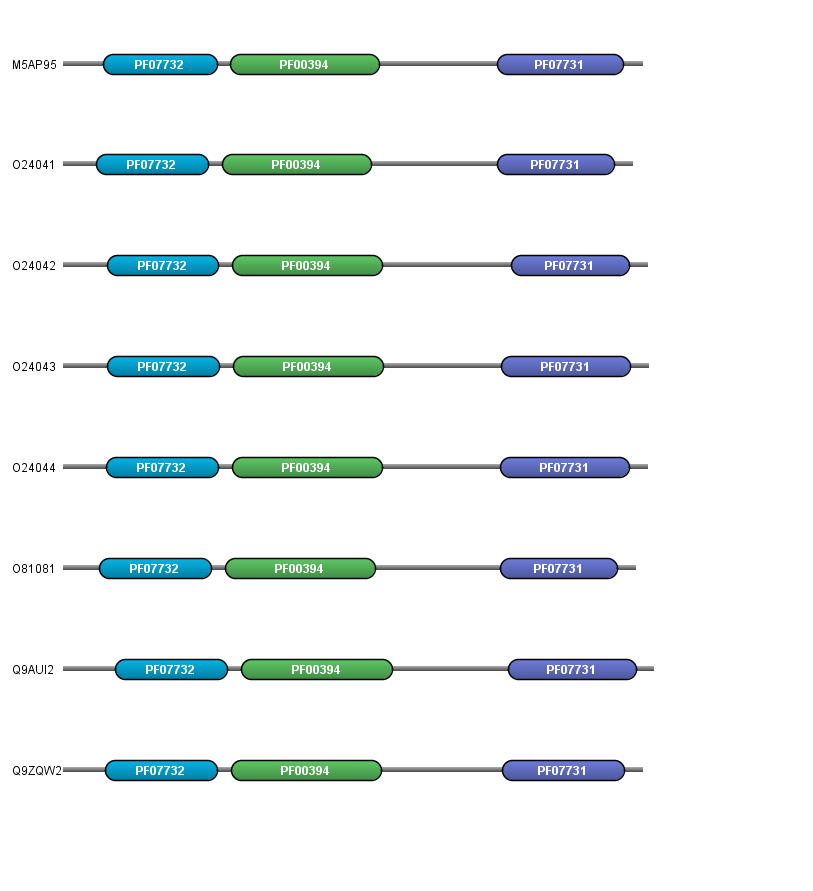


Class-4


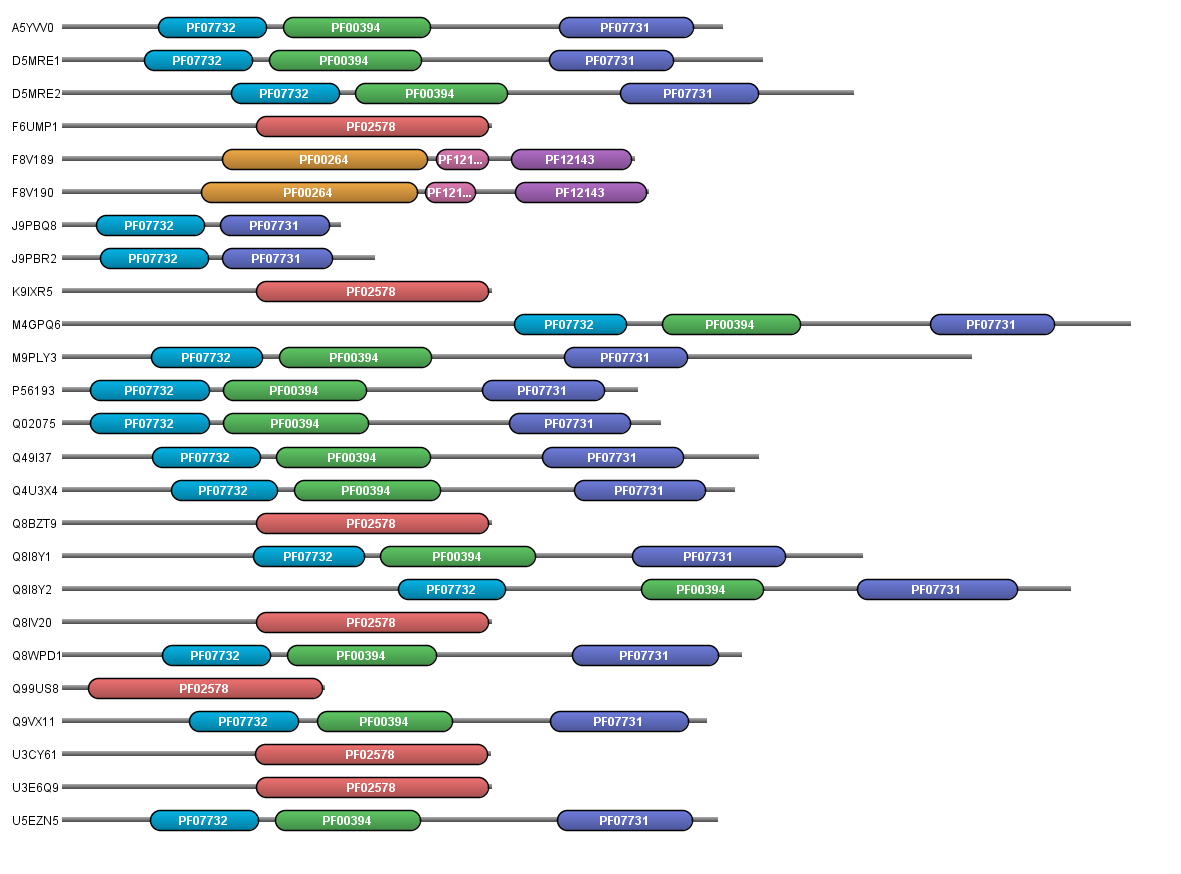


Class-5


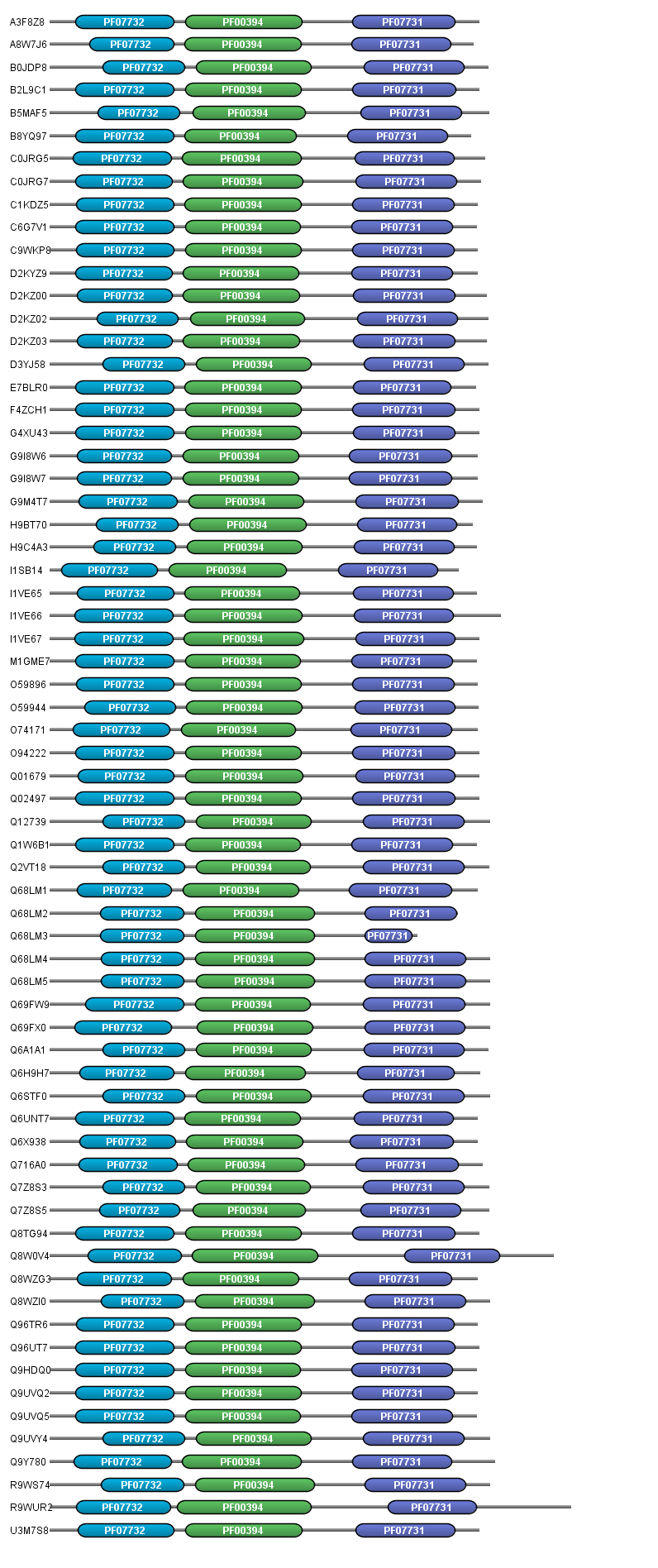


Class-6


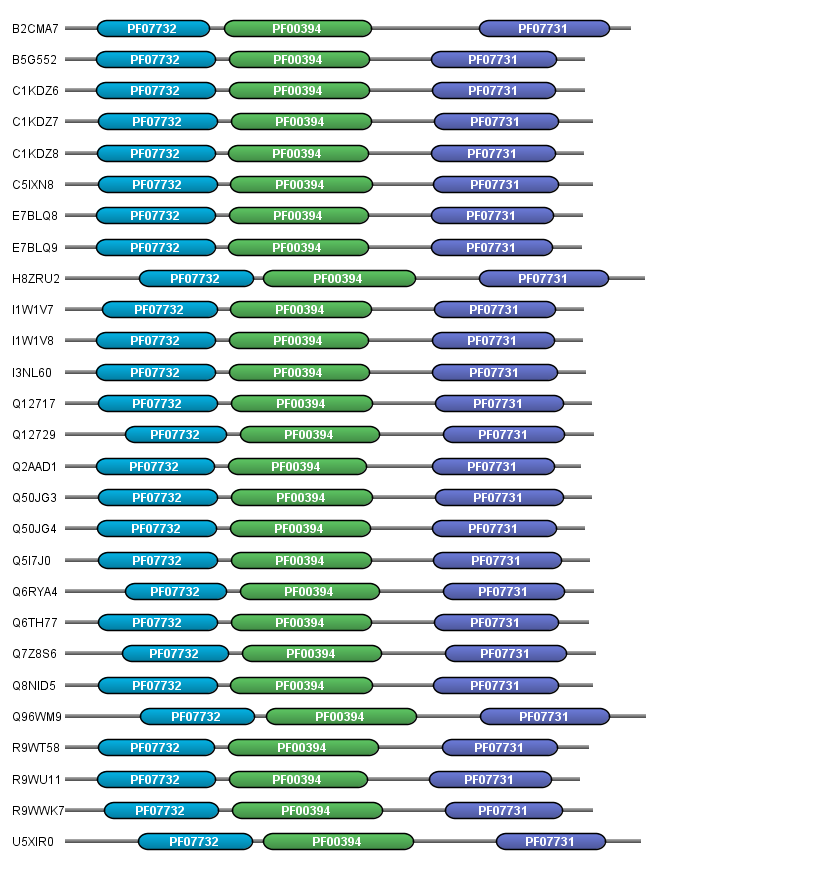


Class-7


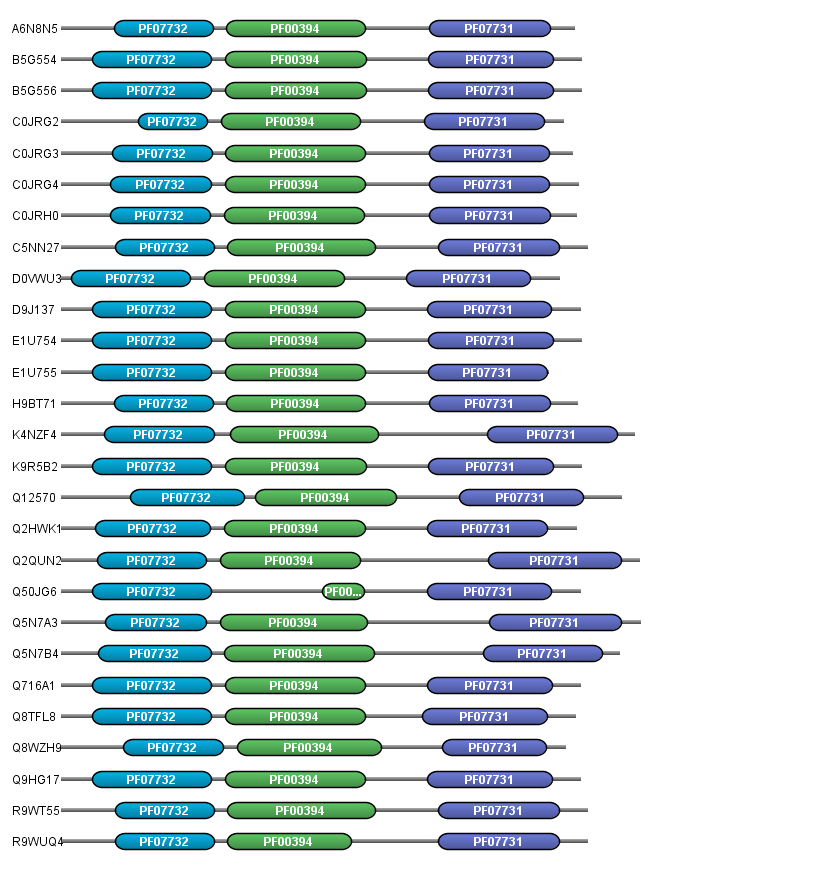


Class-8


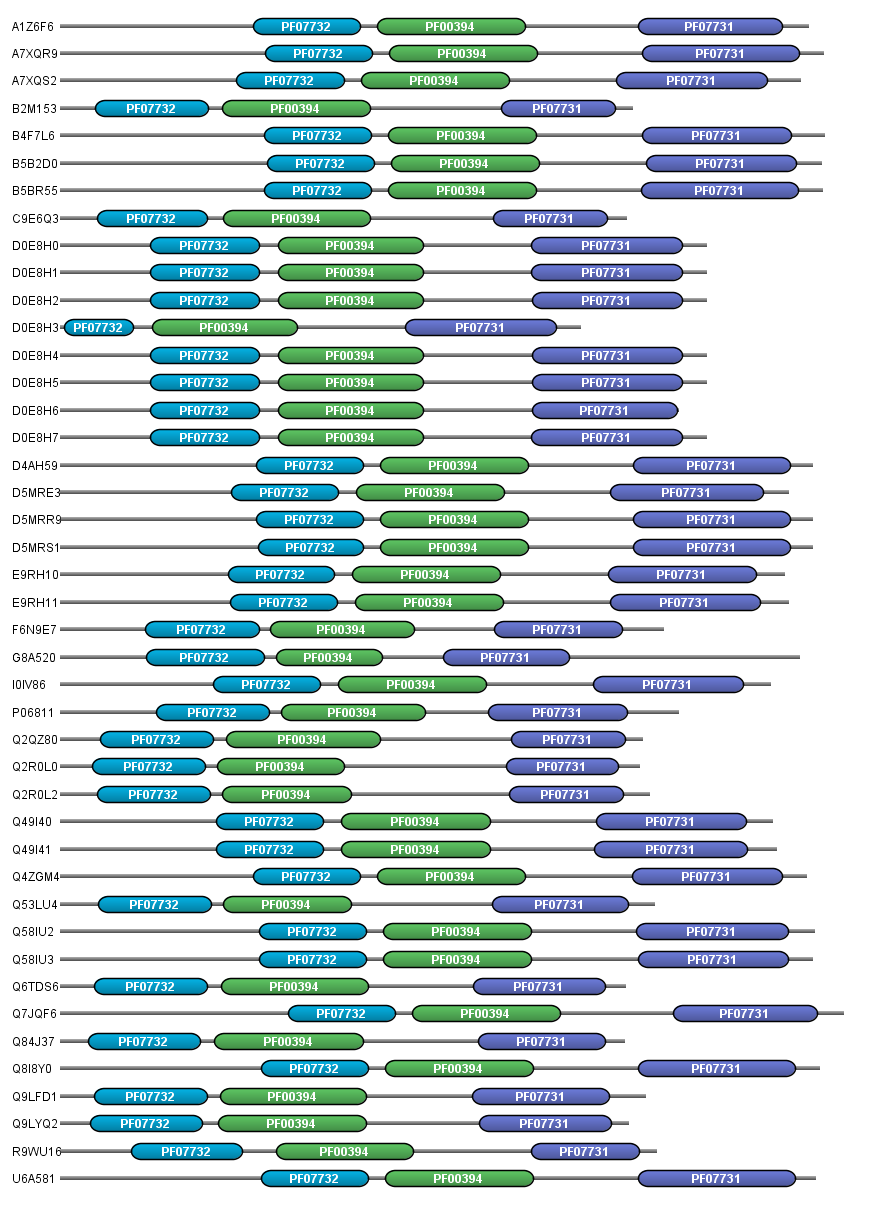


Class-9


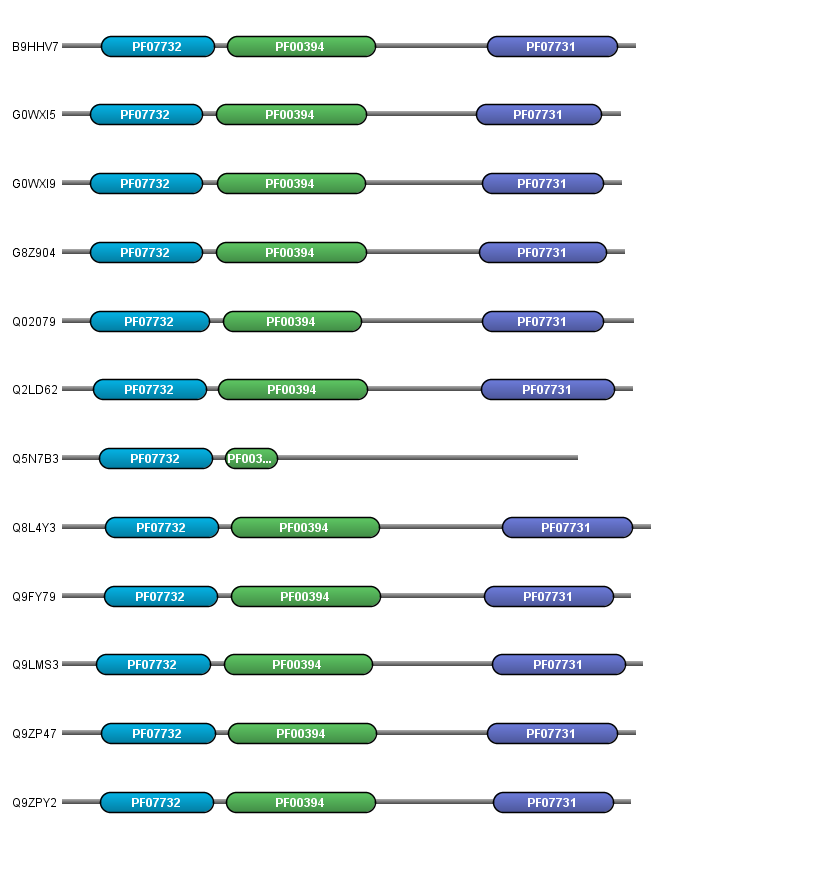


Class-10


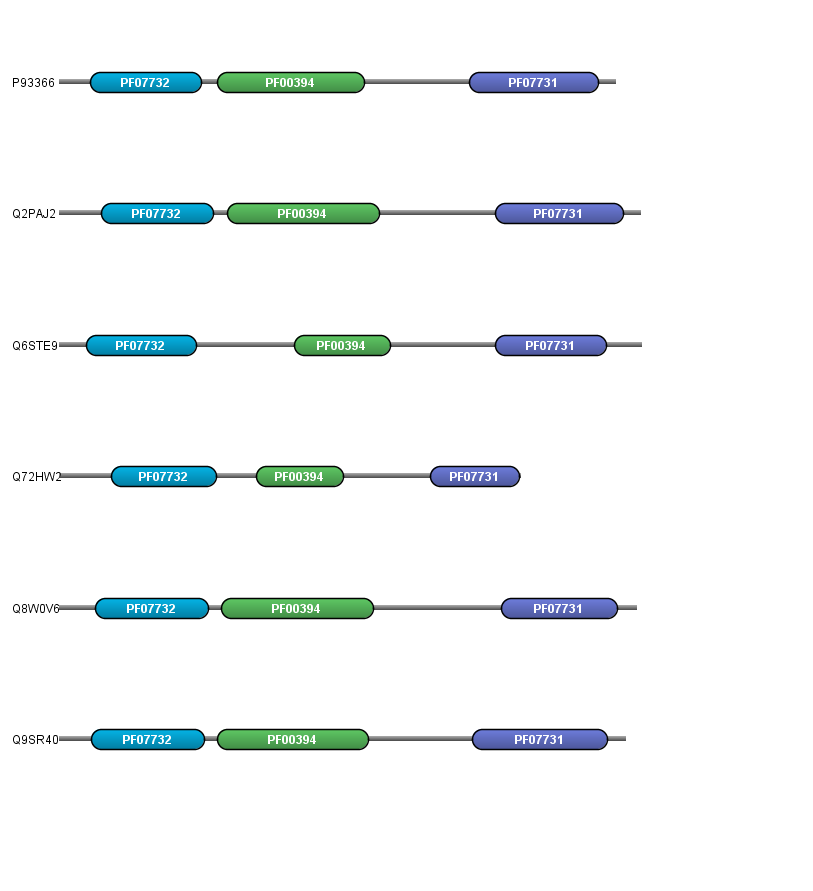


Class-11


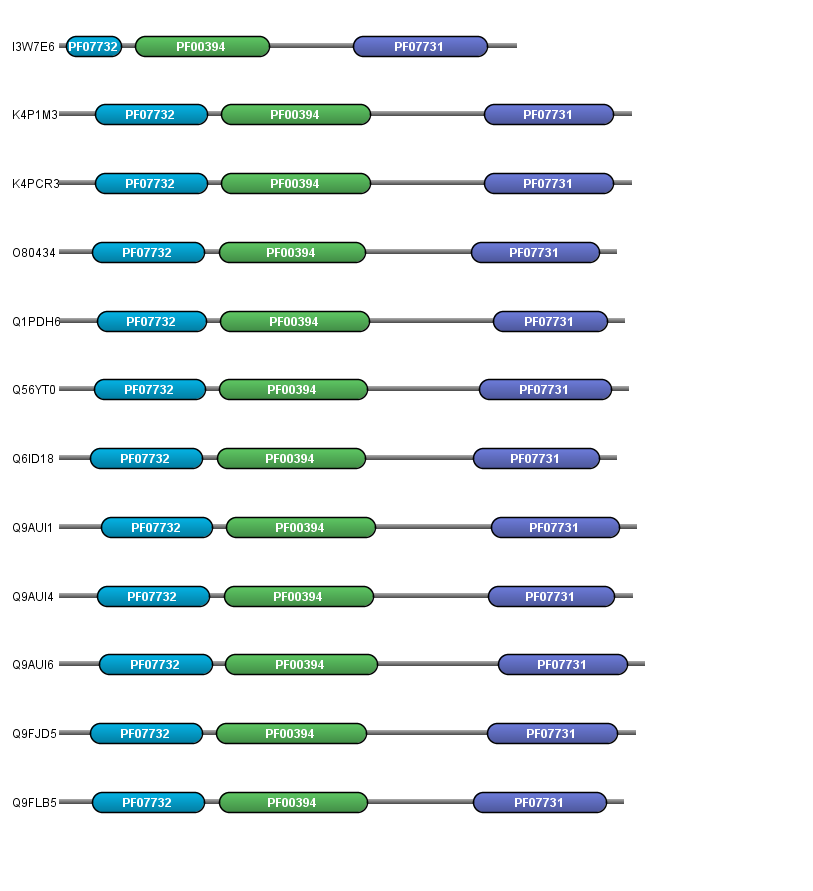

Supplement: Additional file 1 — Domain maps for each of the Laccase subtypes cluster generated using doMosaics (http://www.domosaics.net/). [file 1471-2105-15-S11-S15-S1.docx]
